# On the Spatial Organization of mRNA, Plasmids, and Ribosomes in a Bacterial Host Overexpressing Membrane Proteins

**DOI:** 10.1371/journal.pgen.1006523

**Published:** 2016-12-15

**Authors:** Lieke A. van Gijtenbeek, Andrew Robinson, Antoine M. van Oijen, Bert Poolman, Jan Kok

**Affiliations:** 1 Department of Molecular Genetics, University of Groningen, Groningen, The Netherlands; 2 Zernike Institute for Advanced Materials, University of Groningen, Groningen, The Netherlands; 3 Department of Biochemistry, University of Groningen, Groningen, The Netherlands; CNRS, Toulouse, FRANCE

## Abstract

By using fluorescence imaging, we provide a time-resolved single-cell view on coupled defects in transcription, translation, and growth during expression of heterologous membrane proteins in *Lactococcus lactis*. Transcripts encoding poorly produced membrane proteins accumulate in mRNA-dense bodies at the cell poles, whereas transcripts of a well-expressed homologous membrane protein show membrane-proximal localization in a translation-dependent fashion. The presence of the aberrant polar mRNA foci correlates with cessation of cell division, which is restored once these bodies are cleared. In addition, activation of the heat-shock response and a loss of nucleoid-occluded ribosomes are observed. We show that the presence of a native-like N-terminal domain is key to SRP-dependent membrane localization and successful production of membrane proteins. The work presented gives new insights and detailed understanding of aberrant membrane protein biogenesis, which can be used for strategies to optimize membrane protein production.

## Author Summary

Malfunctioning of particular membrane proteins lies at the heart of some detrimental human diseases. The discovery and correction of bottlenecks in the cellular production of high quantities of membrane proteins for structure/function or drug discovery studies is a huge challenge. Single-cell studies have drastically improved our understanding of microbial physiology but have not been applied yet to protein production. We have studied transcriptional, post-transcriptional, and translational processes in single cells of the protein production host *Lactococcus lactis* and reveal new post-transcriptional obstructions in the synthesis pathway of heterologous membrane proteins. Recalcitrant membrane proteins do not form inclusion bodies but, instead, their transcripts accumulate at the cell poles in severely stressed *L*. *lactis* cells. Importantly, we show that the introduction of a new N-terminal fusion protein that improves recalcitrant membrane protein production coincided with a switch from polar mRNA accumulation to membrane-proximal mRNA localization.

## Introduction

Integral membrane proteins are key elements in essential cellular processes such as signal transmission and membrane transport. Elucidation of their molecular structure is important since their malfunctioning lies at the heart of a great number of diseases and because they constitute important drug targets [1]. Cell-based expression systems are widely employed to produce convenient amounts of recombinant membrane protein for downstream biochemical characterization. In this respect, bacterial species are considered valuable production hosts, next to yeast and insect cells [2–4]. A significant bottleneck in the drug discovery pipeline, however, is that overexpression of membrane proteins in bacterial hosts such as *Escherichia coli* and *Lactococcus lactis* for structural and drug-screening studies remains a significant challenge. Therefore, ample research has aimed to elucidate the physiological responses of these bacteria to overexpression of membrane proteins by means of, for instance, transcriptomic and proteomic analyses in order to identify cellular constraints hampering production [5–7]. Although the ‘omics’ data give a good representation of the average state of the cells in a culture, they do not record problems in membrane protein production at the level of individual cells. Impediments might occur anywhere along the process, from transcription to translation to the insertion of correctly folded proteins in the cytoplasmic membrane. Functional synthesis of most polytopic membrane proteins is dependent on co-translational translocation realized by the signal recognition particle (SRP) pathway, which is responsible for the timely delivery of elongating, membrane protein-producing ribosomes to the membrane [8]. With the rise of techniques to peek inside single cells, the spatiotemporal background of processes that underlie problematic synthesis can now be identified and examined in great detail.

Recently, it was shown in *E*. *coli* that mRNA species encoding SRP-dependent proteins (mostly integral membrane proteins) display translation-dependent membrane localization, including polycistronic mRNAs containing ORFs for both soluble and integral membrane proteins [9–11]. All other mRNAs, including those of the SecB-dependent periplasmic and outer membrane proteins, were maintained throughout the cytoplasm [9]. The SRP-dependent pathway is conserved among all kingdoms of life, but the subset of molecular components deviate between species [12]. The notion that mRNAs of SRP-dependent proteins have a defined spatial organization in bacteria is extremely important in this context. Since codon choice and tRNA-availability differ between cells of prokaryotic or eukaryotic origin, the signal that is responsible for SRP interaction may be unavailable when trying to express a non-optimized gene in an evolutionarily distinct host. It is therefore tempting to speculate that mRNAs, and the heterologous membrane proteins they encode, might not be properly targeted to the membrane due to a lack of the correct bacterial ZIP-code for balanced SRP-dependent membrane delivery.

To address the impact and effect of mRNA targeting on heterologous production of membrane proteins, we investigated the spatial organization of membrane protein-encoding mRNAs in *L*. *lactis* at the single cell level. In contrast to mRNAs of well-expressed membrane proteins that localize at the membrane in agreement with previous studies, our results indicate that poorly expressed membrane proteins experience obstacles during translation, which eventually leads to accumulation of mRNA transcripts at the cell poles. These immobile mRNA clusters are spatially distinct from the location of protein aggregation seeds and from the site where the mRNAs are transcribed. They are also formed when translation is obstructed. Once their disassembly is initiated, a synchronized restoration of translation and growth takes place. Our findings add an empirical understanding of why heterologous membrane proteins are poorly expressed in bacterial hosts, and provide a time-resolved single-cell view on coupled defects in transcription, translation, and growth during aberrant synthesis.

## Results

### Transcripts of poorly expressing membrane proteins accumulate in immobile polar foci

**Fig 1:**
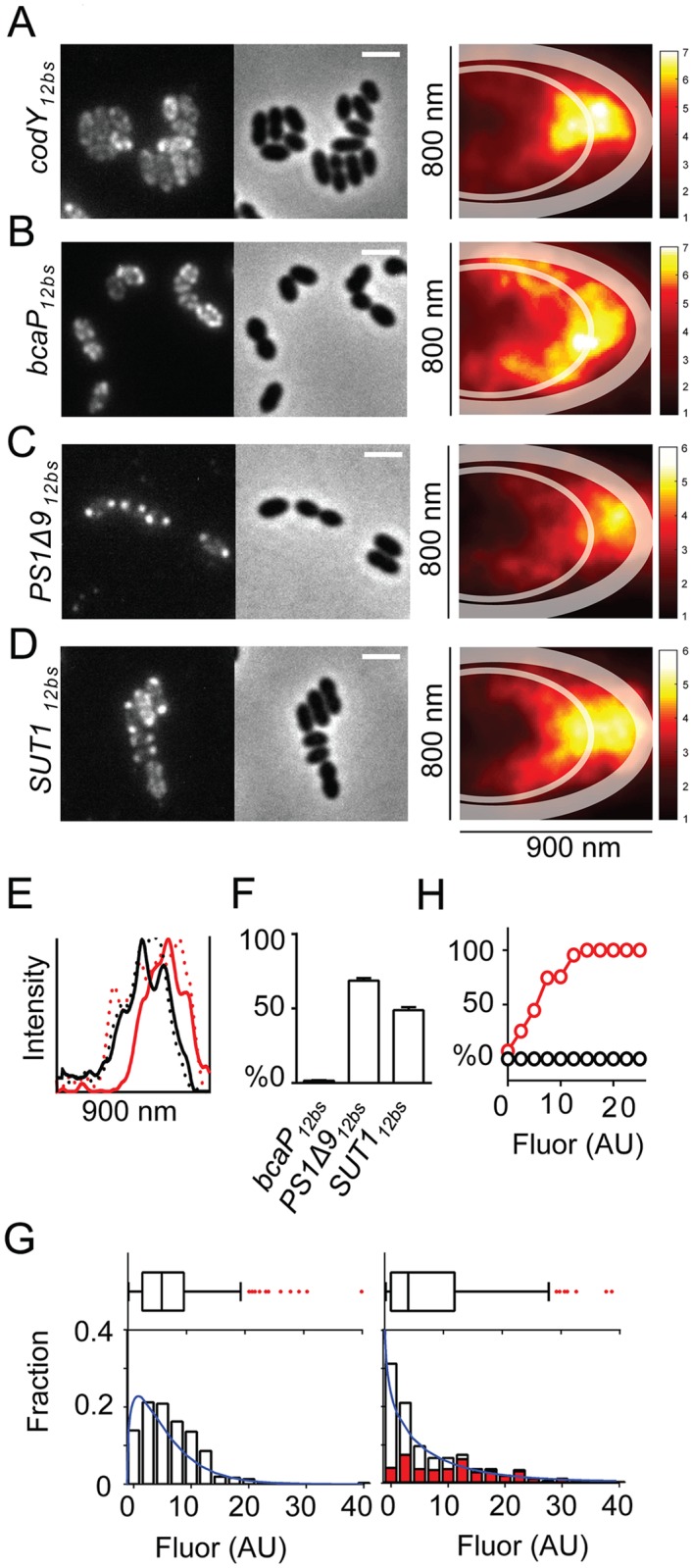
Transcripts of poorly expressing membrane proteins accumulate in immobile polar foci. **(A-D)** Left panels: Localization of *codY*_*12bs*_, *bcaP*_*12bs*_, *PS1Δ9*_*12bs*_ or *SUT1*_*12bs*_ transcripts in *L*. *lactis* NZ9000 cells. Center panels: Corresponding phase contrast images. Scale bar = 2 μm. Colored panels: Location maps constructed from fluorescent TAMRA spots observed in 1185, 877, 765, and 661 cells, respectively, highlighting the preferential localization of each overexpressed mRNA in one half of a model cell. Thick transparent lines: Cell boundaries including the portion occupied by cell wall and membrane as approximated using BcaP-GFP expressing cells (See S1C Fig). Thin transparent lines: Boundaries of chromosomal areas as approximated using DAPI staining in living cells (See S1C Fig). Scale bars depict the relative density of each mRNA species. **(E)** Intensity profiles drawn along the center of the X-axis of the location maps (**(A-D)**, colored panels) of cells expressing *bcaP*_*12bs*_ (solid black line), *codY*_*12bs*_ (dotted black line), *PS1Δ9*_*12bs*_ (solid red line) or *SUT1*_*12bs*_ (dotted red line). **(F)** The percentage of cells with *bcaP*_*12bs*_, *PS1Δ9*_*12bs*_, or *SUT1*_*12bs*_ polar mRNA clusters. Error bars: Standard errors. **(G)** The population-wide distribution of *bcaP*_*12bs*_ or *PS1Δ9*_*12bs*_ mRNA content obtained from single-cell measurements depicted in box plots and histograms (lower panels). Red bars (right panel): The fraction of cells with polar *PS1Δ9*_*12b*_ mRNA clusters. Blue lines: Gamma distributions fitted to expression data. Fluor = fluorescence. **(H)** The percentage of cells containing polar *bcaP*_*12bs*_ (black) or *PS1Δ9*_*12bs*_ (red) mRNA clusters plotted as a function of intracellular TAMRA levels (corresponding to *bcaP*_*12bs*_ or *PS1Δ9*_*12bs*_ mRNA levels).

Differential localization of bacterial mRNAs of SRP-dependent proteins has thus far only been shown to exist in *E*. *coli* [9–11]. Therefore, we first tested, by widefield fluorescence microscopy, whether mRNAs specifying either integral membrane proteins or cytoplasmic proteins adopt different subcellular sites in the Gram-positive bacterium *L*. *lactis*. *L*. *lactis bcaP* mRNA was chosen as a model encoding a well-expressed endogenous amino acid transporter [7,13]. *L*. *lactis codY* mRNA, coding for a central regulator of nitrogen metabolism [13], and *gfp* mRNA were utilized as representatives specifying cytoplasmic proteins. Fluorescence *in situ* hybridization (FISH) was adapted in order to be able to image transcripts with the same probe (*ms2*) by appending the transcripts with repetitive MS2 binding sites (*12bs*) (For validation of the method see S1 Text) [14,15]. The MS2 binding sites were chosen because these also allow for live cell imaging of the transcripts, as discussed below. Since we were specifically interested in the fate of various membrane-protein-mRNAs upon their overproduction, the nisin-induction system for overexpression (NICE) was used to produce recombinant mRNA and cognate proteins [16]. The successful production of STREPII-tagged BcaP and CodY proteins was confirmed by immunoblotting (S1A Fig). After 1 hr of induction with 5 ng/ml nisin, starting at mid-exponential phase, *codY*_*12bs*_ and *gfp*_*12bs*_ mRNAs were mainly detected in the cytoplasm outside the area occupied by the chromosome (Fig 1A and S1B Fig), while the *bcaP*_*12bs*_ transcripts appeared as fluorescent patches along the inside of the cytoplasmic membrane (Fig 1B). We realize that pinpointing the location of overexpressed transcripts does not reflect the native situation. However, the production of recombinant membrane proteins traditionally uses multi-copy number expression vectors. Hence, we wanted to study the temporal and subcellular factors of membrane protein biogenesis in a malfunctioning scenario to understand (over)expression bottlenecks.

Next, we employed FISH to examine the localization of mRNAs encoding two poorly expressed heterologous membrane proteins: A sucrose transporter from *Solanum tuberosum* (SUT1) and the human γ-secretase component, Presenilin 1 missing exon 9 (PS1Δ9). Both proteins are only produced to levels less than 10% of that of BcaP [7,17]. In contrast to *bcaP*_*12bs*_, *SUT1*_*12bs*_ and *PS1Δ9*_*12bs*_ transcripts appeared as single bright fluorescent foci in one or both poles in the majority of cells (Fig 1C and 1D). We ruled out that the polar mRNA foci were methodological artifacts by visualizing untagged *PS1Δ9* and *bcaP* mRNA with gene-specific FISH probes (S1 Text). In order to get a population-wide view of the subcellular distribution of the overexpressed transcripts, >600 randomly chosen cells expressing either *bcaP*_*12bs*_, *SUT1*_*12bs*_, *bcaP*_*12bs*_, or *codY*_*12bs*_ were further examined. Fluorescent spots inside the cells, as well as spot coordinates relative to the cell contour, were obtained using a Gaussian fitting algorithm in the ImageJ ISBatch plug-in [18]. The peak coordinates were normalized, and all spots were jointly projected in one half of a model cell (dimensions: 800×900 nm, based on the average length and width of *L*. *lactis* cells grown in GCDM*; S1C Fig). Location maps of subcellular fluorescence and intensity profiles thereof enabled further discriminating mRNA encoding CodY, BcaP, PS1Δ9, and SUT1 (Fig 1A–1E). *bcaP*_*12bs*_ mRNA is localized along the cytoplasmic membrane, *codY*_*12bs*_ mRNA molecules tend to accumulate at the periphery of the chromosome, while *SUT1*_*12bs*_ and *PS1Δ9*_*12bs*_ mRNA foci are mostly confined to the cell poles. Approximately half of the imaged cells expressing PS1Δ9 or SUT1 contained densely packed, polar mRNA clusters (Fig 1F) marked by spatially confined, yet bright fluorescence (S1D Fig). This type of mRNA-dense spots was far less pronounced or absent in cells expressing *bcaP*_*12bs*_ or *codY*_*12bs*_, respectively (S1D Fig).

In a previous study, no clear differences in the population-averaged levels of transcripts were observed when producing different membrane proteins in *L*. *lactis* [17]. FISH analysis revealed that the population averages of *bcaP*_*12bs*_ and *PS1Δ9*_*12bs*_ transcript levels were roughly similar, but that the distribution of mRNA levels per cell differed quite considerably between the two populations (Fig 1G). After induction, an increase in *bcaP*_*12bs*_ transcript levels was evident when compared to non-induced cells, but this was less so in cells expressing *PS1Δ9*_*12bs*_ mRNA (Fig 1G). Instead, 50% of the latter cells contained very little, or no *PS1Δ9*_*12b*s_ mRNA. In cells with detectable levels of *PS1Δ9*_*12bs*_ mRNA, 90% had polar mRNA clusters (Fig 1G). A small fraction of these cells contained extremely high levels of *PS1Δ9*_*12b*s_ mRNA; cells with high levels of overexpressed mRNA were absent in the *bcaP*_*12bs*_-expressing population. In the remaining ~10% of cells, the *PS1Δ9*_*12b*s_ mRNA localized in a manner similar to that of overexpressed *bcaP*_*12bs*_ transcripts. Higher intracellular levels of *PS1Δ9*_*12b*s_ mRNA correlated with a greater disposition for a cell to carry polar mRNA clusters (Fig 1H). This suggests that there is an expression window for this particular transcript above which cluster formation prevails (mRNA level >10 AU).

### Live cell imaging reveals spatiotemporal aberration of heterologous-membrane-protein mRNA and a decline in transcription

FISH only provides a static view on mRNA localization. We implemented the MS2 system to label mRNAs in living cells to examine mRNA dynamics (For validation of the method see S1 Text) [11,19,20]. In this approach, transcripts extended with MS2 binding sites are specifically recognized by MS2 phage coat proteins fused to GFP. To detect the MS2 binding site-extended transcripts, *L*. *lactis* LG010 was created in which the MS2-GFP protein (MG4: optimized for use in *L*. *lactis*; see S1 Text) can be expressed from the chromosomal *pseudo10* locus [21] using the inducible *nisA* promoter. Importantly, GFP in this study refers to a monomeric superfolder GFP optimized for *L*. *lactis* ([22]; see Materials and Methods). The spatial distribution of the different MS2-GFP-tagged mRNAs was similar to that observed with FISH (S2 Fig). Time-lapse movies revealed that many *bcaP*_*12bs*_ mRNA foci were relatively slow-moving clusters that seemed to be associated with the membrane (S1 Movie). *codY*_*12bs*_ mRNAs diffused as highly dynamic moieties; slow-moving foci similar to those present in *bcaP*_*12bs*_-expressing cells were absent (S2 Movie). *PS1Δ9*_*12bs*_ and *SUT1*_*12bs*_ mRNA formed static and long-lived clusters (>1 hr) at the cell pole (S3 and S4 Movies).

**Fig 2:**
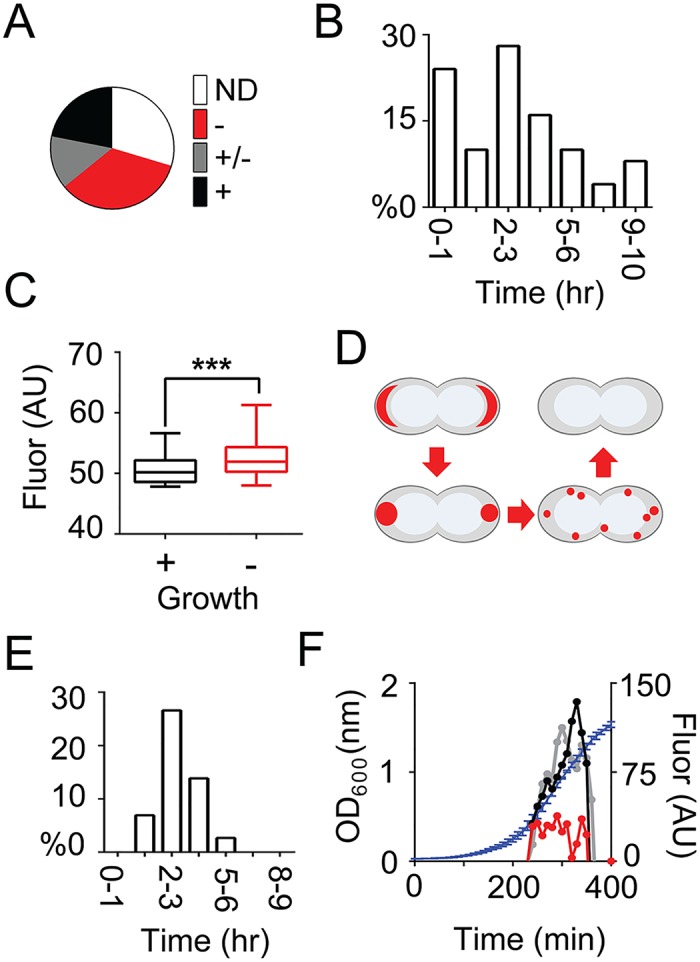
Time-resolved analysis of *PS1Δ9*_*12bs*_ mRNA abundance and localization using time-lapse microscopy. **(A)** *PS1Δ9*_*12bs*_ mRNA expression in *L*. *lactis* LG010 cells was induced following a standard regime after which the cells were transferred to a microscopy slide carrying a thin layer of 1.5% agarose dissolved in GCDM* lacking nisin, for time-lapse fluorescence microscopy analysis. Shown are the portions of 100 tracked cells that contain polar *PS1Δ9*_*12bs*_ mRNA clusters that do not divide after transfer (ND), that grow very poorly and adopt swollen and deviating cell shapes (-), that grow at a moderate rate (+/-) and that grow at a normal rate (+). **(B)** Histogram displaying the time it takes for 100 individual cells to remove MS2-GFP-tagged *PS1Δ9*_*12bs*_ mRNA clusters from their poles, as visualized with time-lapse microscopy. **(C)** Box plots displaying the initial MS2-GFP expression levels, which is directly correlated to the mRNA level, in *PS1Δ9*_*12bs*_-expressing cells that either resumed growth (+) or remained in a non-growing state (-). A Students *t*-test was performed to test for significance (p<0.005). **(D)** Schematic representation of the post-transfer sequence of patterns adopted by MS2-GFP-tagged *PS1Δ9*_*12bs*_ mRNA (colored red). **(E)** Histogram displaying the time it takes for 100 individual cells to stop nisin A-driven transcription of *bcaP*_*12bs*_ mRNA upon transfer of induced liquid cultures to agarose pads with growth medium lacking nisin A, as determined from MS2-GFP distributions in cells using time-lapse microscopy. **(F)** MS2-GFP expression (right axis) monitored in *L*. *lactis* LG010 cells after standard induction, expressing nothing (empty vector control; grey), BcaP (black), or PS1Δ9 (red) and their averaged growth (blue line, left axis). The MS2-GFP fluorescence values are normalized for OD_600_.

Growth of, and mRNA localization in *PS1Δ9*_*12bs*_-expressing cells were followed over time using MS2-GFP (MG4). One-third of the cells did not grow and polar foci remained visible for up to 4 hrs (S5 Movie and Fig 2A and 2B). In another population tertile, polar foci redistributed into multiple smaller foci synchronously with a switch from prolonged (~3 hrs) growth arrest to cell expansion with disturbed division (S6 Movie and Fig 2A and 2B). The last fraction contained less intense fluorescent foci, an indication that P_*nisA*_-induced expression had been relatively low in these cells, that disappeared within 1 hr, after which growth continued as normal (S7 Movie and Fig 2A and 2B). Hence, only mild expression at the initiation of the time-lapse experiment led to microcolony formation after removal of polar mRNA clusters (Fig 2C). We identified a clear sequence of post-transfer *PS1Δ9*_*12bs*_ mRNA patterns (S5 and S6 Movies and Fig 2D). First, mRNA formed crescent patches at the poles. Then, a redistribution to spots with Gaussian distributions occurred. These spots eventually fell apart into multiple smaller foci and ultimately disappeared. Under identical conditions, *bcaP*_*12bs*_-expressing cells did not cease to grow, and P_*nisA*_-driven expression continued for 60–90 min after transfer from liquid medium to microscopy slides lacking the inducer nisin (S8 Movie and Fig 2E).

Using MS2-GFP, we consistently observed that there are at least two deviant subpopulations of *PS1Δ9*_*12bs*_-expressing cells: Non-growing cells with mRNA clusters and dividing cells without any visible *PS1Δ9*_*12bs*_ mRNA. Presumably, the latter cells grew because they were either not (yet) induced or already had terminated P_*nisA*_-controlled transcription. P_*nisA*_-driven co-expression of MS2-GFP with *PS1Δ9*, but not with BcaP, decreased without affecting the population-wide growth (Fig 2F). Hence, either transcription from at least the *nisA* promoter or translation in general is abrogated in *L*. *lactis* cells producing recalcitrant membrane proteins. In any case, both scenarios create a negative feedback that contributes to reduced production yields.

### Polar mRNA clusters are spatially unrelated to protein aggregation seeds

**Fig 3:**
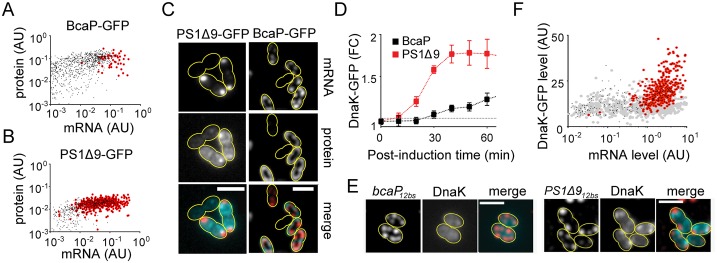
Polar mRNA clusters are spatially unrelated to protein aggregation seeds. **(A)** Single-cell *bcaP-gfp*_*12bs*_ mRNA content plotted as a function of intracellular BcaP-GFP protein abundance. Red encircled dots: Cells with polar *bcaP-gfp*_*12bs*_ clusters. **(B)** Single-cell *PS1Δ9-gfp*_*12bs*_ mRNA content plotted as a function of intracellular PS1Δ9-GFP protein abundance. Red encircled dots: Cells with polar *PS1Δ9-gfp*_*12bs*_ clusters. **(C)** Co-visualization of *bcaP-gfp*_*12bs*_ or *PS1Δ9-gfp*_*12bs*_ mRNA (FISH) with their protein products. Bottom panels: false-colored overlays of mRNA (red) and protein (cyan). Images were subjected to deconvolution to enhance the contrast. **(D)** Time course of fold changes (FC) of DnaK-GFP expression in *L*. *lactis* LG029, a strain in which *dnaK* was replaced with *dnaK-gfp*, after induction of expression of PS1Δ9 (red squares) or BcaP (black squares) compared to control cells (dashed grey line). **(E)** Co-visualization of DnaK-GFP and overexpressed *PS1Δ9*_*12bs*_ or *bcaP*_*12bs*_ mRNA in *L*. *lactis* LG029. Right panels: False colored-overlays of DnaK-GFP (cyan) and mRNA (red). **(F)** DnaK-GFP levels in single *L*. *lactis* LG029 cells displayed as a function of levels of *bcaP*_*12b*s_ mRNA (grey filled circles) or *PS1Δ9*_*12bs*_ mRNA (black dots). Black dots encircled in red: Cells with polar *PS1Δ9*_*12bs*_ clusters. Scale bar = 2 μm. Yellow lines indicate cell contours.

To examine if polar mRNA clusters correspond to sites of protein aggregation, BcaP and PS1Δ9 were fused to GFP. Even though GFP fused to the extra-cytoplasmic PS1Δ9 C-terminus likely interferes with its membrane insertion, information can still be gained on completion of translation, protein targeting, and protein-mRNA co-localization. The cells were induced to express BcaP-GFP or PS1Δ9-GFP for 1 hr, after which the level and subcellular localization of each of the GFP-tagged proteins and their respective mRNAs were examined using FISH. Cells with polar *bcaP-gfp*_*12bs*_ mRNA clusters (<1% of the population) contained the highest amount of *bcaP-gfp*_*12bs*_ transcripts, and held relatively high levels of BcaP-GFP (Fig 3A). The production window for *bcaP*_*12bs*_, defined as the range of mRNA levels that do not lead to mRNA clusters, was significantly wider than that of *PS1Δ9*_*12bs*_ mRNA (Fig 3B). PS1Δ9-GFP was clearly detected albeit at levels at least an order of magnitude lower than BcaP-GFP (Fig 3A–3C). No reports on inclusion bodies in *L*. *lactis* exist, but misfolded membrane proteins are known to co-fractionate with the membrane proteome [23]. In agreement, we occasionally observed membrane-proximal fluorescent protein aggregates, even in BcaP-GFP-expressing cells (S3A Fig). The protein aggregates did not typically co-occur with polar clusters of *bcaP-gfp*_*12bs*_ mRNA. When present in the same cell, they did not co-localize (S3A Fig). No signs of PS1Δ9-GFP membrane insertion were detected. Rather, PS1Δ9-GFP was present as aggregation seeds [24] at the membrane (Fig 3C and S3B Fig). Of the larger PS1Δ9-GFP aggregates, the majority (90%) did not co-localize with, but was often found in the proximity of polar mRNA foci (Fig 3C).

We attempted to indirectly detect (aggregates of) truncated or incompletely translated fusion proteins by localizing the molecular chaperone DnaK. DnaK associates with protein aggregates and inclusion bodies in *E*. *coli* [24,25]. Levels of DnaK-GFP were significantly elevated 15 min after initiation of *PS1Δ9*_*12bs*_ expression (Fig 3D). The cellular distribution of DnaK-GFP was examined in combination with FISH to visualize *PS1Δ9*_*12bs*_ or *bcaP*_*12bs*_ mRNA. The distribution of DnaK-GFP inside *bcaP*_*12b*s_ expressing cells was similar to that of DnaK-GFP in control cells (Fig 3E and S3C Fig). Clusters of DnaK-GFP only formed in PS1Δ9-expressing cells, but hardly ever at the pole(s), where *PS1Δ9*_*12bs*_ mRNA gathers (Fig 3E and S3C Fig). Rather, DnaK-GFP localized to the most intense PS1Δ9-GFP protein foci: DnaK foci are on average a similar distance away from polar *PS1Δ9*_*12bs*_ mRNA clusters as were the PS1Δ9-GFP foci (S3D Fig). Location maps of PS1Δ9-GFP and DnaK-GFP foci show that they have very comparable intracellular distributions, which are quite different from that of *PS1Δ9*_*12bs*_ mRNA (S3E Fig). Although no overlap in localization between polar *PS1Δ9*_*12bs*_ mRNA clusters and DnaK-GFP was observed, elevated levels of DnaK-GFP are correlated with the presence of polar *PS1Δ9*_*12bs*_ mRNA clusters, indicating that those cells are, or have been, under severe stress (Fig 3F).

### Plasmids predominantly co-localize with the nucleoid, not with mRNA-dense polar clusters

**Fig 4:**
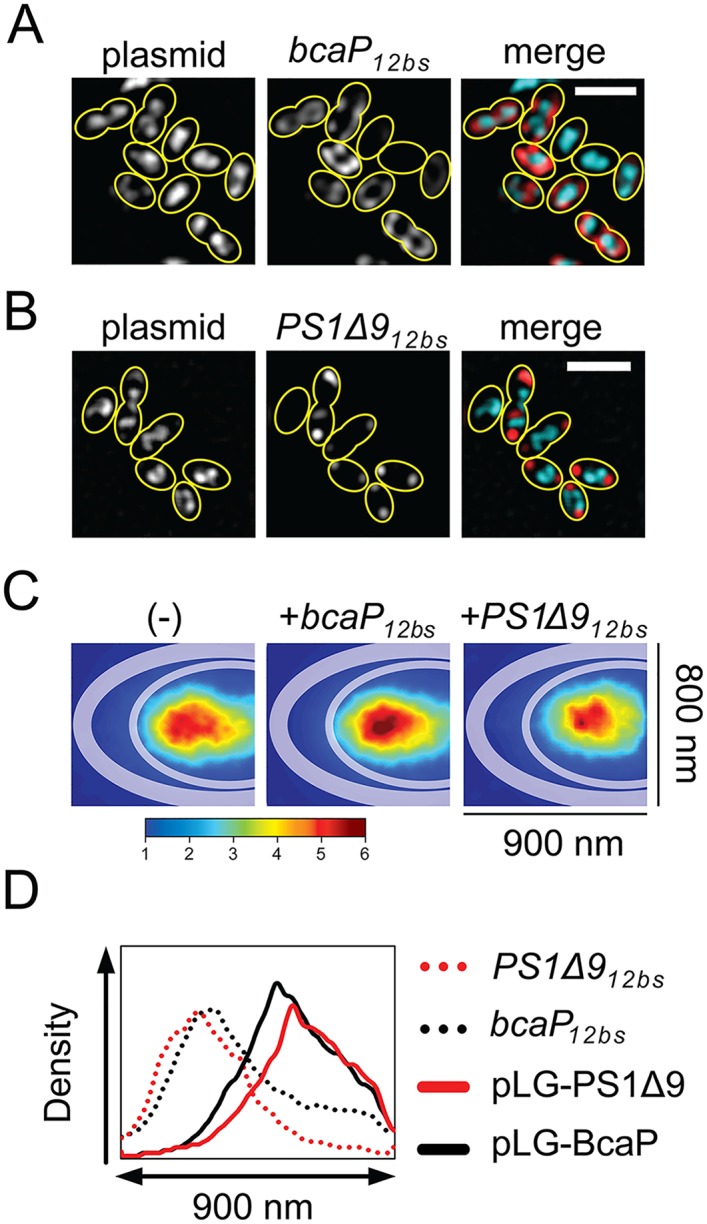
pNZ8048 plasmids predominantly localize in the chromosomal area, not with mRNA-dense polar clusters. Plasmids tagged with *parS*/ParB-GFP were co-visualized with overexpressed *bcaP*_*12bs*_ mRNA **(A)** or *PS1Δ9*_*12bs*_ mRNA **(B)** using FISH. Right panels show false-colored images in which mRNA and plasmids are represented in red and cyan, respectively. Images were subjected to deconvolution to enhance the contrast. Scale bar = 2 μm. Yellow lines indicate cell contours. **(C)** ParB-GFP in all of the captured cells (± 200 cells per experiment) were traced with the ImageJ PeakFinder plug-in and jointly projected into one half of a model cell as described earlier. The intracellular distribution of ParB-GFP foci under various gene expression scenarios (empty vector control (-), BcaP or PS1Δ9 expression) is represented as location maps to highlight the predominant sites of plasmid localization averaged over all cells. **(D)** Intensity profiles of overexpressed mRNA and co-visualized plasmids created from the location maps to illustrate differences in distribution of pLG-BcaP (black solid line), pLG-PS1Δ9 (red solid line), *bcaP*_*12bs*_ (black dotted line), and *PS1Δ9*_*12bs*_ (red dotted line) along the X-axis of the model cells. Scale bars in microscopy images represent 2 μm.

We entertained the possibility that pNZ8048 plasmids are predominantly present in the cell pole(s) like other multi-copy number plasmids, which might seed the formation of foci for mRNAs of poorly expressed membrane proteins due to high local transcription [26–28]. Plasmid pNZ8048 was visualized using a Gram-positive ParB/*parS* system (M. Kjos, J. Siebring, J.W. Veening, personal communication) and DNA FISH. A *parS* site was introduced in pNZ8048, pLG-BcaP and pLG-PS1Δ9, and *L*. *lactis* LG045a was constructed, a strain that constitutively produces low levels of ParB-GFP (the *parS* binding protein fused to GFP). The *L*. *lactis* NZ9000 genome does not contain a ParA homolog [29], allowing visualization of the subcellular location of *parS*-labeled pNZ8048 derivatives (Fig 4 and S4 Fig).

ParB-GFP expressed in cells carrying pNZ8048 without a *parS* sequence distributed homogeneously (S4A Fig). Unlike some high-copy plasmids in other bacterial species [26–28], pNZ8048 primarily populates the area occupied by the chromosome in *L*. *lactis* (S4 Fig). Co-visualization of the plasmids and the full-length transcripts they specify shows that they are spatially separated (Fig 4A, 4B and 4D). Since our plasmid imaging approach does not have the time-resolution required to monitor rapid spatiotemporal changes, we cannot rule out the possibility that transertion, the coupled transcription, translation and translocation of membrane proteins, does not occur here [30]. Adding nisin to induce expression of *bcaP*_*12bs*_ or *PS1Δ9*_*12bs*_ transcripts did not severely affect the location of pNZ8048-derived plasmids, but location maps of plasmids show that induction of PS1Δ9 caused the area occupied by plasmids to slightly shrink compared to control cells (Fig 4C and 4D).

Because polar clustering of *PS1Δ9*_*12bs*_ transcripts cannot be explained by the spatial distribution of pNZ8048, we reasoned that downstream processes, including targeting of mRNA for degradation or complications in synthesis of PS1Δ9 might be involved. The shrinkage of the area occupied by the plasmids (and nucleoid) suggests that transertion of PS1Δ9 is inhibited under these conditions.

### The relationship between mRNA cluster formation, translation activity, and mRNA degradation

**Fig 5:**
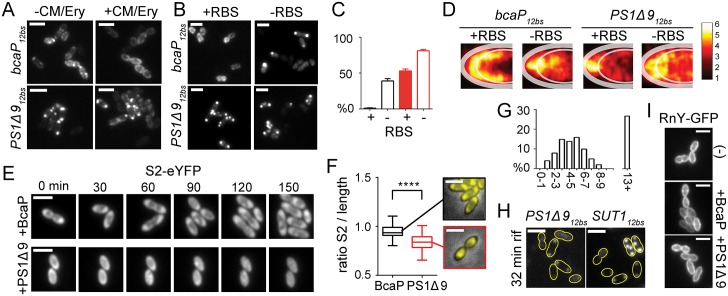
Effect of polar clustering and ribosome binding on degradation and localization of overexpressed mRNAs. **(A)** *L*. *lactis* LG010 cells with MS2-GFP-tagged *bcaP*_*12bs*_ or *PS1Δ9*_*12bs*_ mRNA treated or not with Cm and Ery. **(B)** FISH images of *L*. *lactis* NZ9000 cells expressing *bcaP*_*12bs*_ or *PS1Δ9*_*12bs*_ mRNAs with or without functional RBS, and visualized by *ms2* probe hybridization. **(C)** The percentage of cells (n = 500) expressing *bcaP*_*12bs*_ or *PS1Δ9*_*12bs*_ with (+) or without (-) RBS and containing mRNA clusters. Error bars = standard errors. **(D)** Location maps constructed from FISH images of 500 cells showing the predominant distribution of *bcaP*_*12bs*_ or *PS1Δ9*_*12bs*_ with or without RBS. **(E)** Snap shots of time-lapse microscopy of *L*. *lactis(rpsB*::*rpsB-eYFP)* cells expressing *bcaP*_*12bs*_ or *PS1Δ9*_*12bs*_. **(F)** Boxplots of the ratio between the S2-eYFP protein spread along the long cell axis and total cell length obtained from 100 cells expressing BcaP or PS1Δ9, as well as representative pictures of the corresponding S2-eYFP distributions. ***: A Student’s *t*-test was performed to test for significance (p<0.005). **(G)** Time (hr) required for individual *PS1Δ9*_*12bs*_-expressing cells (n = 100) to restore growth after transfer to agarose pads lacking nisin, as determined by time-lapse microscopy. **(H)** Examples of *PS1Δ9*_*12bs*_ and *SUT1*_*12bs*_ transcripts remaining in cells after 32 min treatment with rifampicin. **(I)** RnY-GFP localization in fixed *L*. *lactis* LG024a cells expressing nothing (-), *bcaP*_*12bs*_, or *PS1Δ9*_*12bs*_. Strain LG024a constitutively expresses *rnY-gfp* ectopically from its own promoter. The fusion gene was introduced additional to *rnY* to not interfere with essential mRNA breakdown.

What is the effect of translation on the localization and degradation of the studied mRNAs? Are polar mRNA clusters still available for translation or could they correspond to bacterial analogs of dedicated mRNA degradation hubs that are found in eukaryotic cells during stress? In *E*. *coli*, treatment with translation-inhibiting kasugamycin results in a loss of membrane-localized mRNAs [9]. We observed the same with overexpressed MS2-GFP-visualized *bcaP*_*12bs*_ mRNAs when *L*. *lactis* cells were treated with translation-inhibiting antibiotics erythromycin (Ery) and chloramphenicol (Cm) (Fig 5A). The bulky MS2-labeled mRNAs did not enter the nucleoid region due to chromosome condensation induced by Ery/Cm treatment [31–33] (S5A Fig). In contrast to *bcaP*_*12bs*_, *PS1Δ9*_*12bs*_ mRNA remained visible in the poles after antibiotics treatment. These structures apparently do not readily release transcripts upon inhibition of translation. To further investigate the effect of translation on mRNA localization, we mutated the *aaggagg* ribosomal binding site (RBS) on *gfp*_*12bs*_ transcripts by changing it to *aattcgg* (S5B Fig), and employed the same strategy to disrupt the RBS on *bcaP*_*12bs*_ and *PS1Δ9*_*12bs*_. Using FISH, we observed that a high percentage of the cells expressing RBS-less *bcaP*_*12bs*_ or *PS1Δ9*_*12bs*_ mRNA contained transcript clusters (Fig 5B and 5C). This suggests that the punctuate foci correspond to mRNAs that are blocked from translation. Closer examination (Fig 5D) of the population-wide localization of the RBS-less mRNAs revealed that they cluster more closely to the nucleoid (and thus also to the expression plasmids), while the RBS-containing *PS1Δ9*_*12bs*_ mRNA clusters are predominantly present at the cell poles (see also Fig 1C).

The localization of ribosomes in *L*. *lactis* is similar to that in other bacteria: The major fraction of the S2-eYFP protein—and therefore ribosomes—localizes outside the nucleoid [32,34,35] (Fig 5E and S5C Fig). Time-lapse movies similar to those described above reveal that non-growing cells expressing PS1Δ9 undergo a reorganization of S2-eYFP localization from typically nucleoid-occluded to highly homogeneously distributed (Fig 5E). Since 70S ribosomes are not able to freely diffuse into the nucleoid [32,34,35], a homogenous distribution indicates a loss of 70S ribosomes, and thus of translation. Non-induced cells transferred to agarose slides lacking nutrients showed a similar phenotype, suggesting that the loss of 70S ribosomes overlaps with entry into a stationary-like growth phase (S5C Fig). Surprisingly, S2-eYFP is excluded from the poles in PS1Δ9-expressing cells (Fig 5F), which indirectly suggests that polar *PS1Δ9*_*12bs*_ mRNA clusters are not accessible for ribosomes. The finding that Ery/Cm treatment does not lead to a release of mRNA from polar clusters strengthens this supposition. Under similar conditions, in about the same time required for cells to get rid of polar MS2-labeled *PS1Δ9*_*12bs*_ clusters (Fig 2B), S2-eYFP localization and growth were restored to wild-type (Fig 5G).

Determining the lifetimes of mRNAs might help understanding the cause of the polar mRNA accumulation observed here. We treated cells expressing different transcripts and cognate proteins with rifampicin to stop translation, and used FISH to follow the level of each mRNA at several time points after rifampicin addition. We used FISH instead of Northern hybridization to obtain a population-wide quantifiable view on mRNA degradation. Thus, we were able to distinguish subpopulations of cells with significantly altered mRNA degradation parameters (see below). Considerable amounts of *PS1Δ9*_*12bs*_ (t_½_ = 10.9 min) and *SUT1*_*12bs*_ (t_½_ = 19.7) transcripts were still present after a 32-min treatment with rifampicin, in contrast to *codY*_*12bs*_ (t_½_ = 7.1 min) and *bcaP*_*12bs*_ (t_½_ = 6.2 min) (Fig 5H and S5D Fig). The degradation rate of *codY*_*12bs*_ and *bcaP*_*12bs*_ were in agreement with those reported previously (S5E Fig) [36], which shows that mRNA degradation rates in *L*. *lactis* are generally lower than those measured in other fast-growing bacterial species such as *E*. *coli* and *B*. *subtilis* [37–39]. Despite considerable deviations between experimental replicates, the half-lives of *PS1Δ9*_*12bs*_ and *SUT1*_*12bs*_ transcripts did not differ substantially from the estimated median half-life of all mRNAs in *L*. *lactis* (t_½_ = 10.9 min; S5E Fig). Separation of cells into populations that did or did not contain polar mRNA clusters, and recalculation of their half-lives, revealed that, in cells without polar mRNA clusters, *PS1Δ9*_*12bs*_ and *SUT1*_*12bs*_ mRNA is degraded at much higher rates than similar mRNA trapped inside polar mRNA clusters (*PS1Δ9*_*12bs*_: t_½_ = 4.6 min and t_½ (cluster)_ = 16.1 min; *SUT1*_*12bs*_: t_½_ = 6.2 min and t_½ (cluster)_ = 22.4 min; S5F Fig). Degradation of overexpressed *PS1Δ9*_*12bs*_ or *bcaP*_*12bs*_ mRNA lacking an RBS takes place at a higher rate (t_½_ = ~2.5 and ~1.7 min, respectively) than the original transcripts (t_½_ = ~10.9 and ~ 6.2 min, respectively; S5G Fig). The fact that the RBS-less *PS1Δ9*_*12bs*_ and *bcaP*_*12bs*_ mRNAs yielded nearly identical degradation curves indicates that the mRNA half-life of at least these two mRNA species is mainly influenced by ribosome occupancy in *L*. *lactis*. These results also imply that the observed slow degradation of *PS1Δ9* mRNA does not originate from intrinsic signals in the alien mRNA sequence, but depends on binding of ribosomes and/or downstream processes. Importantly, the positioning of the major ribonuclease in Gram-positive bacteria, membrane-tethered RNase Y [40], remains unaffected by *PS1Δ9*_*12bs*_ or *bcaP*_*12bs*_ overexpression (Fig 5I).

### The presence of an endogenous N-terminal domain is key to SRP-dependent membrane localization

**Fig 6:**
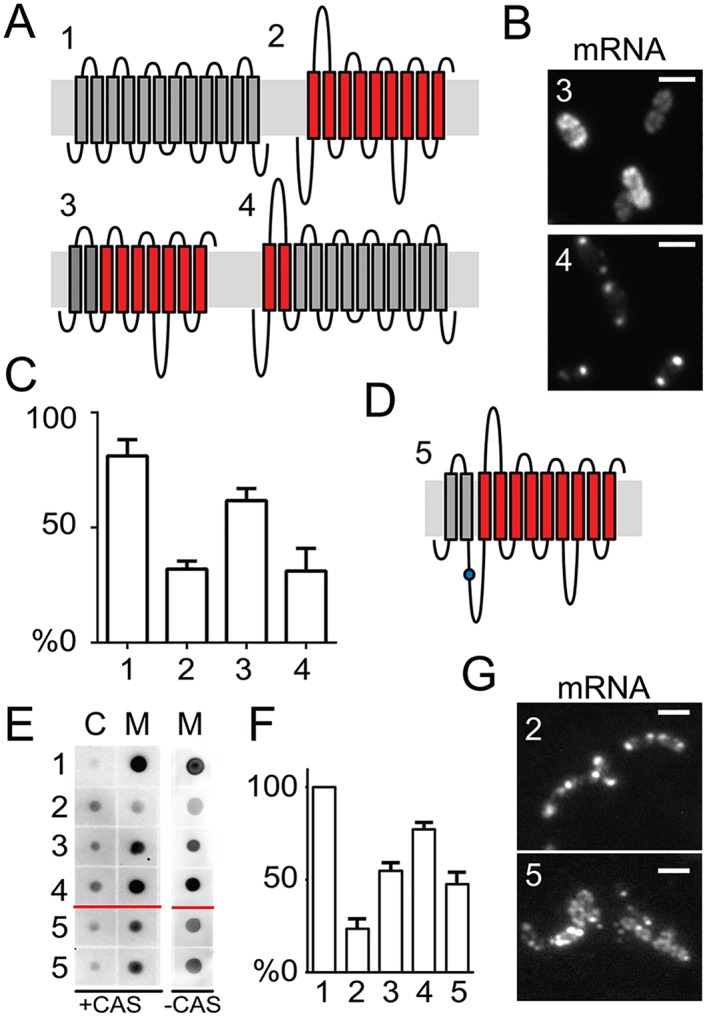
N-terminal domain of membrane proteins influences mRNA localization and protein production. **(A)** Schematic representation of the transmembrane domains, as predicted by TMHMM, of BcaP (1) and PS1Δ9 (2) and chimeric proteins in which a portion of N-terminal domains of PS1Δ9 (PS1Δ9^N^) is replaced by those of BcaP (3) and vice versa (4). **(B)** mRNA localization in representative cells expressing *bcaP*^*N*^-*PS1Δ9*_*12bs*_ (3) or *PS1Δ9*^*N*^*-bcaP*_*12bs*_ (4) transcripts. **(C)** The percentage of cells (n = 400) without polar mRNA of the proteins depicted in A. **(D)** Schematic representation of PS1Δ9 (red bars) fused via its N-terminal domain and a flexible linker containing a TEV protease site (blue dot) to the newly developed fusion partner BLS (for BcaP Leading Segment; grey bars). **(E)** Dotblots of cytoplasmic fractions (C) and membrane fractions (M) of *L*. *lactis* NZ9000 cells grown in GCDM* or GCDM* supplemented with 1.5% casitone, and expressing the proteins shown in A and D. **(F)** Quantification of dotblot signals. Averaged signals are displayed as percentages of the signals obtained for BcaP expression. **(G)** Representative image of cells expressing *PS1Δ9*_*12bs*_ or *BLS-PS1Δ9*_*12bs*_ mRNA as visualized by FISH. Numbers in B, C, E, and F refer to the construct numbers in A and D.

Could a defect in proper targeting of mRNA-ribosome complexes to the membrane play a role the mislocalization of the recombinant mRNAs? It is well known that SRP-dependent targeting is important for co-translational translocation, and that recognition of a membrane protein emerging from the ribosomes by SRP is dependent on signals located on the N-terminal domain of SRP-dependent proteins [9,41]. Optimization strategies by which slow codons are incorporated at specific sites might stimulate timely SRP recognition [42], and fusion tags can improve the guiding or initial insertion of heterologous RNC-ribosome-mRNA complexes to the membrane [43–45]. To test if the N-terminal domain of PS1Δ9 (PS1Δ9^N^) is less adequate for membrane-targeting in *L*. *lactis* than that of BcaP (BcaP^N^), we swapped both regions, including the first two transmembrane domains, and re-examined their mRNA localization (Fig 6A and 6B). Indeed, a high number of *bcaP*^*N*^-*PS1Δ9*_*12bs*_ transcripts was now localized at the membrane instead of in polar clusters (Fig 6B and 6C). Vice versa, *PS1Δ9*^*N*^*-bcaP*_*12bs*_ mRNAs were mainly found in polar clusters (Fig 6B and 6C). Overexpression of the corresponding proteins was monitored using quantitative blotting, in which the membrane protein fractions of *L*. *lactis* cells were collected and directly spotted onto nitrocellulose membranes. In agreement, the replacement of the N-terminal domain of PS1Δ9 with that of BcaP dramatically elevated production yields, while the presence of the N-terminal PS1Δ9 domain lowered BcaP production (Fig 6E and 6F).

The finding that polar mRNA accumulation of *PS1Δ9*^*N*^*-bcaP*_*12bs*_ had decreased while the corresponding protein production had improved prompted us to design a general optimization strategy for improved production of recalcitrant membrane proteins. The same N-terminal domain of BcaP was now fused to the N-terminus of PS1Δ9 via a flexible linker that included a TEV protease cleavage site (Fig 6D). We coined this new fusion partner BcaP leading segment (BLS). To test the effectiveness of BLS on PS1Δ9 production, the various *L*. *lactis* NZ9000 strains were grown in two types of media: GCDM* and GCDM* supplemented with extra peptides through the addition of casitone. The latter has been shown to positively affect the production of membrane proteins [46]. Induction was performed according to the standard regime, after which the quantity of original and chimeric membrane proteins in the membrane fraction was examined as described above. In both types of media an increased signal of BLS-PS1Δ9 over PS1Δ9 was observed, indicating that production of PS1Δ9 had improved (Fig 6E and 6F). Moreover, mRNA of BLS-PS1Δ9 was more often observed at the membrane than clustered in the cell poles (Fig 6G). Although follow-up studies are required to address the quality of overexpressed BLS-PS1Δ9, we conclude that the BLS-tag has great potential as a fusion partner to elevate the levels of recalcitrant (heterologous) membrane proteins in *L*. *lactis* by guiding their insertion in the cytoplasmic membrane.

### Membrane protein mRNAs of the AT-rich *L*. *lactis* contain an increased U-density over other mRNAs

**Fig 7:**
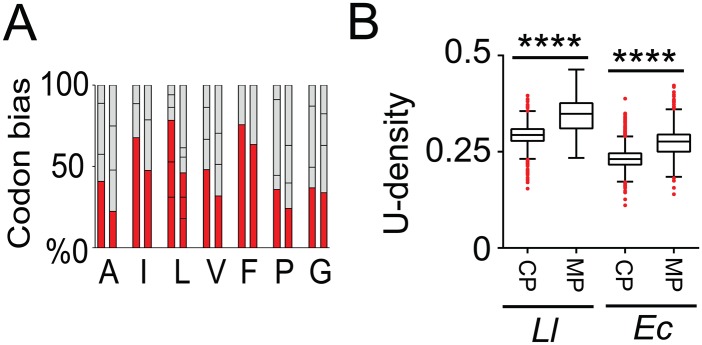
Increased uracil density in *L*. *lactis* genes encoding membrane proteins. **(A)** The relative codon bias for all hydrophobic amino acids in *L*. *lactis MG1363* (left bars) and *E*. *coli K12* (right bars). The red bars indicate the codon(s) with the highest uracil content. **(B)** Boxplots show that the uracil density of transcripts encoding membrane proteins is increased in both species. A Student’s *t*-test was performed: the uracil density was significantly different (p < 0.0001; indicated by ****) between the two types of transcripts for both *E*. *coli* and *L*. *lactis*.

Thus far, we were able to show a subset of cellular consequences of polar mRNA clusters, and excluded the localization of plasmids, protein aggregates, the chaperone DnaK, and the main constituent of the RNA degradosome, RNase Y, as a direct cause for cluster formation. What then, besides ribosome exclusion, could drive the predisposition of mRNAs of recalcitrant membrane proteins at the poles? Codons for hydrophobic amino acid residues that constitute the transmembrane domains of membrane proteins have a strong uracil (U) bias [47]. This is more pronounced in bacteria such as *E*. *coli* than in higher, eukaryotic cells through differences in codon choice. The increased U-density of membrane protein mRNA was suggested to function as a post-transcriptional zip code for membrane targeting. Since *L*. *lactis* is an AT-rich organism, the general U-density of transcripts is intrinsically higher than that of *E*. *coli*. If the U-density of transcripts is of evolutionary importance, we would expect to see an adapted, higher U-bias in the preferred codons specifying hydrophobic amino acid residues in *L*. *lactis*. This is indeed the case for hydrophobic amino acid residues in *L*. *lactis*, whereas this bias is less pronounced in *E*. *coli* (Fig 7A). Therefore, despite the AT-rich genomic sequence of *L*. *lactis*, the U-density of membrane protein-encoding mRNA is significantly higher than that of mRNAs of cytosolic proteins, but also than that of membrane protein-encoding mRNAs of *E*. *coli* (Fig 7B). This suggests that in AT-rich organisms the deployment of mostly relatively U-rich codons for hydrophobic amino acid residues has been selected for, through which the relative difference between the general U-density of mRNAs encoding membrane or cytoplasmic proteins is maintained.

## Discussion

*L*. *lactis* has emerged as a suitable host for membrane protein production, although expression of heterologous membrane proteins is still suboptimal. To gain insights into potential obstacles, we examined the fate of transcripts of well and poorly expressing membrane proteins, and the relationship between mRNA localization, transcription, translation, and degradation. In *E*. *coli*, transcripts for many integral membrane proteins are present in SRP-dependent co-translational translocation complexes during the major part of their lifetime [9]. Transcripts encoding the homologous transporter BcaP localized to the membrane in *L*. *lactis* while disruption of their translation led to loss of membrane-proximal mRNA foci, suggesting that a similar transertion system operates in this bacterium. Transcripts of poorly expressed membrane proteins, *H*. *sapiens* PS1Δ9 and *S*. *tuberosum* SUT1, formed distinctive polar clusters in *L*. *lactis*. Importantly, neither the expression vector pNZ8048 nor protein aggregates co-localized at the poles. Although high-copy number plasmids have been localized predominantly at the cell poles of bacteria [26–28], pNZ8048-derivatives randomly distributed in the cells, with a predisposition in the area where the chromosomal DNA is also situated. The ColE1-derived plasmid pBluescript, with ~50 copies per cell, does not adopt any specific subcellular location in *E*. *coli* [48]. Both this and our study show that high-copy number plasmids do not necessarily cluster at the poles.

The occurrence of polar mRNA clusters was translation-dependent, positively correlated to the level of heterologous transcripts, and was always accompanied by severe growth defects, loss of nucleoid-occluded ribosomes, and activation of the heat-shock response. Furthermore, PS1Δ9-expressing cells displayed confined plasmid distribution and delayed degradation of polar *PS1Δ9*_*12bs*_ mRNA clusters. The disappearance of polar mRNA foci was a prerequisite for cells to be able to grow again, and was accompanied by a loss of nisin-driven transcription but also by relocalization and increase of ribosomal protein S2-eYFP. The sudden release of many *PS1Δ9*_*12bs*_ transcripts from polar foci, however, severely impacted growth, often leading to defects in cell division. All of these observations are an indication that cells with polar mRNA clusters adopt an altered cellular state. Previous ensemble studies of *L*. *lactis* overproducing membrane proteins suggested that the stringent response might be involved [17]. High alarmone ((p)ppGpp) levels in *E*. *coli* and *B*. *subtilis* lead to a shift from transcription of *rRNA* genes to transcription of genes involved in nutrient generation, preparing the cell for survival in a nutrient-poor environment [49,50]. In *Staphylococcus aureus*, (p)ppGpp inhibits ribosome assembly by binding GTPases involved in the maturation of ribosomal subunits [51]. It is therefore tempting to speculate that the loss of active ribosomes in *L*. *lactis* is triggered by the stringent response due to nutrient limitation or expression of the heterologous protein PS1Δ9.

The presence of polar mRNA clusters correlated to cells with a strong induction, as evidenced by both the level of overexpressed mRNAs and co-expressed MS2-GFP. As with the *E*. *coli* Walker strains, in which T7 RNA polymerase-driven transcription is reduced compared to the parent strain [52,53], the synthesis of less mRNA might improve the production of recalcitrant membrane proteins in *L*. *lactis*. Lowering the inducer concentration would be a straightforward solution albeit that nisin A-driven expression triggers a heterogeneous response at doses below 0.5 ng ml^-1^ [54]. Interestingly, mutations in the sensor histidine kinase NisK led to a 2- to 8-fold improvement in membrane protein production due to an increase in leaky expression in *L*. *lactis* DML1-3 strains [23]. We can now assume that this background transcription causes a steady state production of low levels of recombinant mRNA that do not lead to mRNA clustering or proteotoxic stress, prior to induction of gene expression.

The question remains what exactly causes the mRNA cluster formation. Below, three scenarios are discussed.

### Availability for ribosomes

Overexpressed mRNAs encoding soluble proteins were mainly present in the nucleoid-free space, whereas RBS-less transcripts accumulated at the boundaries of the chromosomal area instead of the poles. A model on the spatial organization of intracellular mRNA localization in bacteria predicts that a great portion of transcripts reside in the nucleoid-free space and that a cyclic pattern of ribosome flow occurs, in which ribosomes that have finished translation in the nucleoid-free space pick up new transcripts from the nucleoid [55]. Transcripts might thus not readily diffuse away from their site of production without effective binding by ribosomes, resulting in the cluster formation at the nucleoid periphery observed here. Importantly, the adaxial clusters of untranslatable *bcaP*_*12bs*_ or *PS1Δ9*_*12bs*_ transcripts resembled polar mRNA clusters in terms of density, while S2-eYFP was excluded from the poles whenever PS1Δ9 was produced. Abrogated translation of *PS1Δ9*_*12bs*_ transcripts might therefore be the cause of their polar clustering.

### Uracil-density

In agreement with a previous study in *E*. *coli* [47], the uracil density of membrane protein-encoding mRNA in the AT-rich *L*. *lactis* is significantly higher than that of mRNAs of cytosolic proteins. The fact that, through codon bias, this uracil-richness has been preserved in bacteria strengthens the idea that it might play a role in membrane protein biogenesis. Recently, two cold-shock proteins, CspE and CspC, were shown to interact with uracil-rich transcripts and the inner membrane of *E*. *coli* [56]. This suggests that these CSPs not only stabilize transcripts during cold shock, but also deliver specific U-rich cargos to the cytoplasmic membrane. Remarkably, gene expression of most CSPs in *L*. *lactis* was downregulated upon PS1Δ9 expression but not upon BcaP-expression [7,17]. Because *bcaP* mRNA (0.36) and other endogenous membrane protein transcripts contain a higher U-density than the heterologous *PS1Δ9* (0.29) and *SUT1* (0.31) mRNA, it is tempting to hypothesize that the homologous *bcaP*_*12b*s_ mRNA is recognized (better) by *L*. *lactis* homologs of *E*. *coli* CspE and CspC, such as CspE, CspB, and CspD2. This is supported by the notion that even RBS-less *PS1Δ9*_*12bs*_ transcripts are still more prone to accumulate in dense clusters than RBS-less *bcaP*_*12bs*_ transcripts, the latter of which might be pulled away from the cell center, resulting in fewer mRNA clusters. If CSPs exert similar functions in *L*. *lactis* and *E*. *coli*, the decline in these membrane-targeting factors observed during PS1Δ9-expression would lead to a loss of membrane-proximal mRNA localization, and possibly to polar predisposition instead.

### SRP recognition

When SRP recognition and delivery fails, synthesis of membrane proteins continues in the hydrophilic cytoplasm, leading to protein misfolding and aggregation [12]. A consensus SRP-specific signal peptide on integral membrane proteins has never been identified, but recent data suggest that translational pausing at mRNA elements 30–40 codons downstream of the codons that specify the SRP binding site triggers SRP-binding [41,57]. Neither the gene encoding PS1Δ9 nor that for SUT1 was codon-optimized for *L*. *lactis*. Due to differences in codon choice and tRNA-availability, *cis*-acting signals that promote SRP-binding might be absent when trying to express a non-optimized gene in an evolutionarily distinct host [41,57]. The idea that the timely targeting of ribosomes to the membrane by SRP constitutes a bottleneck during membrane protein overproduction has been explored in *E*. *coli* [58,59]. Competition between binding to the ribosome-nascent chain of trigger factor (TF), a folding chaperone for cytoplasmic proteins, and SRP influences efficient targeting of membrane proteins [59]. An increase in correctly inserted membrane proteins was established by decreasing the amount of TF while at the same time elevating the SRP level. Some N-terminal fusion proteins are known to improve membrane protein production, putatively through promoting SRP-dependent targeting and/or membrane insertion of the overexpressed protein [43,60–62]. We show here that these adaptations could also be responsible for guiding ribosomes carrying alien mRNA to the membrane: Interchanging the N-terminal domains of PS1Δ9 and BcaP resulted in a reversal in mRNA localization patterns and improved production of PS1Δ9. We delivered a proof-of-principle by introducing the BcaP N-terminal domain as a new *L*. *lactis*-specific fusion partner, BLS, which improves the heterologous production of recalcitrant membrane proteins such as PS1Δ9.

## Methods

### Bacterial strains, media and culture conditions

Bacterial strains and plasmids used in this study are listed in S1 Table. For cloning purposes, *Lactococcus lactis* NZ9000 was grown as a standing culture at 30°C in Difco M17 medium (BD, Franklin Lakes, NJ, USA) containing 0.5% (w/v) glucose (GM17). When required, chloramphenicol and/or erythromycin were added at a final concentration of 5 μg ml^-1^ or 3 μg ml^-1^, respectively. For all other analyses, *L*. *lactis* was grown in chemically defined medium (CDM; pH 6.8) with 0.5% glucose [63] lacking riboflavin (GCDM*), in which it reaches a growth rate of ~0.55 h^-1^. When stated, 1.5% (w/v) pancreatic digest of casein (Bacto Casitone; BD Biosciences, Franklin Lakes, NJ, USA) was added to GCDM* Chemically defined SA medium with 0.5% (w/v) glucose and 20 μg ml^-1^ 5-fluoro-orotic acid (5-FOA; Sigma-Aldrich, St. Louis, MO, USA) as a sole pyrimidine source was used for the generation of chromosomal knock-ins, as described previously [64]. *Escherichia coli* DH5α (Life Technologies, Gaithersburg, MD, USA) was used for cloning purposes; it was grown aerobically at 37°C in LB medium (Formedium, Norfolk, UK) with, when required, erythromycin or ampicillin at a final concentration of 150 or 100 μg ml^-1^, respectively. All antibiotics were obtained from Sigma-Aldrich.

### Recombinant DNA techniques and oligonucleotides

Standard molecular cloning techniques were performed essentially as described [65]. Chromosomal DNA from *L*. *lactis* was isolated using the Wizard Genomic DNA Purification Kit (Promega Life Sciences, Madison, WI, USA). Plasmids and PCR products were isolated and purified using the High Pure Plasmid Isolation and PCR Purification kit (Roche Applied Science, Mannheim, Germany) and the NucleoSpin Gel and PCR Clean-up kit (Macherey-Nagel, Düren, Germany) according to the manufacturers’ instructions. Enzymes were purchased from Fermentas (Thermo Fisher Scientific Inc, Waltham, MA, USA) and New England Biolabs (Ipswich, MA, USA). PCR reactions were performed with Phusion or DreamTaq polymerase (both from Fermentas) according to the manufacturer’s protocol. In case DNA fragments were prepared for uracil-excision DNA-based engineering, PfuX7 polymerase was employed (Morten HH Nørholm, DTU, Denmark) [66]. The obtained PCR fragments were mixed and treated with the USER enzyme mixture (New England Biolabs), yielding 9–12 nt-long overhangs annealing to complementary overhangs. No ligation was required, and USER-treated mixtures were directly used to transform *L*. *lactis* or *E*. *coli*. Oligonucleotides employed in this study are listed in S2 Table and were purchased from Biolegio BV (Nijmegen, The Netherlands). Competent *E*. *coli* cells were transformed using heat-shock, while electrocompetent *L*. *lacti*s cells were transformed using electroporation with a Bio-Rad Gene Pulser (Bio-Rad Laboratories, Richmond, CA). All nucleotide sequencing was performed at Macrogen Europe (Amsterdam, The Netherlands).

### Plasmid and strain construction

Nucleotide sequences of the primers and their role in the construction work described below are presented in S1 Text and S2 Table. Pertinent regions of all plasmids were sequenced to confirm their proper nucleotide sequences. All plasmids and strains that were used or constructed during this study are listed in S1 Table.

### Protein analysis, western blotting, and immunodetection

*L*. *lactis* NZ9000 carrying pLG-BcaP or pLG-CodY was inoculated 1:100 in chemically-defined medium containing 0.5% (w/v) glucose (GCDM; (18)) and grown as standing culture at 30°C to an OD_600_ of 0.4, and induced with 5 ng ml^-1^ of nisin A (Sigma-Aldrich). Cells were harvested 2 hrs post-induction by centrifugation at 9,000×*g* for 15 min at 4°C, washed with ice-cold 100 mM potassium phosphate (KPi) pH 7.0 and resuspended to OD_600_ of 1 in ice-cold 100 mM KPi. For lysis, glass beads were added to 1 ml of the cell suspension, and cells were broken by 3×35 sec incubations in a mini-bead beater (Biospec Products, Bartlesville, OK) with 1-min intervals of cooling at 4°C. Cell debris was removed by centrifugation at 7650×*g* for 15 min (4°C). To detect overexpressed StrepII-tagged BcaP, membrane fractions were collected by high-speed centrifugation (267,000×*g* for 30 min at 4°C) in an Optima TLX Ultracentrifuge (Beckman Coulter, Brea, CA, USA). For immunodetection of StrepII-tagged BcaP, membranes were washed with 10 mM KPi buffer containing 10% glycerol, resuspended in 2×SDS loading buffer (1M Tris-HCl, pH 6.8, 10% SDS, 20% (v/v) glycerol, 25% (v/v) β-mercaptoethanol, 0.05% bromophenol blue), boiled for 5 min, subjected to SDS-(12.5%) PAGE. For immunodetection of StrepII-tagged CodY, cleared cell lysates were mixed with 2×SDS loading buffer, boiled for 5 min, and separated by SDS-(12.5%) PAGE. Proteins were transferred by semi-dry blotting to a polyvinylidene difluoride membrane (Roche Applied Sciences). For dot-blotting, strains were grown and fractionated as described above. Membrane and cytoplasmic proteins were directly spotted onto a nitrocellulose membrane (0.45 μm, Bio-Rad). Immunodetection of transferred or spotted StrepII-tagged proteins was performed as follows. Membranes were washed with 1×TBS-T (20 mM Tris, 150mM NaCl, and 0.1% Tween 20, pH 7.5) blocked with 5% bovine serum albumin in TBS-T, blocked with 0.001% avidin in 1×TBS-T for 20 min, washed with 1×TBS-T, after which the proteins were detected with *Strep*MAB-Classic (IBA, Göttingen, Germany), using Amersham anti-mouse polyclonal antibodies from sheep, conjugated to horseradish peroxidase and ECL detection (both from GE Healthcare Europe GmbH, Eindhoven, The Netherlands) according to manufacturers’ instructions.

### Preparation of cells for *in vivo* fluorescence microscopy imaging or RNA FISH

Generally, *L*. *lactis* was grown overnight in GM17 with appropriate antibiotics prior to microscopy analysis. Overnight cultures were washed in sterile 1×PBS (137 mM NaCl, 2.7 mM KCl, 10 mM Na_2_HPO_4_, 1.8 mM KH_2_PO_4_, pH 6.8) and inoculated 1:100 in GCDM*, to limit background fluorescence, and antibiotics when required. Cultures were grown to mid-exponential phase (equivalent to OD_600_ of ~0.4) and induced with nisin A at a final concentration of 5 ng ml^-1^. Depending on the experiment, cells were incubated for another 0 to 90 min.

For *in vivo* fluorescence microscopy imaging, 0.4 μl of a culture was transferred onto a microscopy slide that was prepared as follows: Multitest-slides (MP Biomedical, Santa Ana, CA, USA) were cleaned using 5M KOH and MilliQ, after which they were coated with 1.5% agarose (Sigma-Aldrich) dissolved in fresh GCDM*.

To stop translation, both chloramphenicol and erythromycin were added to an induced culture, each to a final concentration of 200 μg ml^-1^. Cultures were then incubated for another 30 min, after which cells were examined using fluorescence microscopy (see below).

To determine the degradation rate of overexpressed transcripts, the mRNAs were produced for 1 hr in the presence of 5 ng ml^-1^ of nisin A. Then rifampicin was added to a final concentration of 200 μg ml^-1^ to inhibit transcription. Cells were fixed (see below) at different time points after rifampicin addition (0, 1, 2, 4, 8, 16, 32, and 64 min) and prepared essentially as described in the following paragraph. This procedure was repeated at least twice for each mRNA on separate days. The final mRNA half-lives were determined as further described in **Data analysis**.

For RNA FISH, performed essentially as described [14] and adapted for *E*. *coli* cells [15], the cells were fixed by directly adding formaldehyde to the cultures to a final concentration of 3.7%. After incubation for 30 min at RT, the treated cells were washed twice with dyethylpyrocarbonate (DEPC)-treated 1×PBS, resuspended in 1×PBS containing 10 mg ml^-1^ lysozyme (Sigma-Aldrich), incubated for 30 min at 37°C, washed twice with 1×PBS and permeabilized in 70% ethanol. Permeabilized cells were washed twice with RNA FISH wash buffer (40% formaline and 2×SSC (150 mM NaCl, 15 mM sodium citrate, pH 7.0) in DEPC-treated MilliQ), resuspended in RNA FISH hybridization buffer (10% (w/v) PEG6000 (Sigma-Aldrich), 2 mM vanadyl ribonucleoside complex (VRC, 200 mM; New England Biolabs), 1 mg ml^-1^ yeast RNA, 40% formaline, and 2×SSC in DEPC-treated MilliQ) containing 1 mM of each FISH probe and incubated for 16 hrs in a water bath at 30°C. Prior to imaging, excess probe was removed by two washings with 200 μl RNA FISH wash buffer. Finally, the cells were resuspended in 2×SSC and transferred to a microscopy slide coated with 1.5% agarose in 1×PBS.

For DNA FISH, the cells were treated similar to preparation for RNA FISH, except for the following changes. An RNase (Roche) step (50 μg ml^-1^, 1 hr, and 37°C) was incorporated after permeabilization with ethanol and cells were washed twice with 1×PBS. Prior to 16-hrs probe hybridization step, cells were heated to 78°C for 10 min in hybridization buffer, after which the TAMRA-labeled *ms2* probe was added to a final concentration of 1 mM.

The oligonucleotide probes used are summarized in S3 Table. All probes were purchased from Biolegio BV and were covalently linked at both ends to carboxytetramethylrhodamine (TAMRA) dyes. The *ms2* FISH probe was designed to specifically hybridize to each of the 12 MS2 binding sites fused to the 3’-ends of overexpressed transcripts, leading to a theoretical 24-fold amplification of the signal. The three *bcaP*-FISH or *PS1Δ9*-FISH probes were designed to anneal next to each other and to target the middle of the protein-coding region of the transcripts.

### Fluorescence microscopy

All micrographs were obtained with a DeltaVision Elite inverted epifluorescence microscope (Applied Precision, GE Healthcare, Issaquah, WA, USA) equipped with a stage holder, a climate chamber, a seven-color combined set InsightSSI Solid-State Illumination module, and an sCMOS camera (PCO AG, Kelheim, Germany). A 100× phase-contrast objective (NA 1.4, oil-immersion, DV) was used for image capturing, in combination with SoftWorX 3.6.0 software (Applied Precision) to control the microscope setup and to perform single-time-point or time-lapse imaging of cells. The following standard fluorescence filter sets were used for visualization: GFP, excitation at 475/28 nm and emisson at 525/48 nm; TAMRA/TRITC, excitation at 542/27 nm and emission at 597/45 nm; DAPI, excitation at 390/18 nm and emission at 435/48 nm (all using a polychroic beam splitter suitable for DAPI, FITC, TRITC, Cy5); eYFP, excitation at 438/24 nm, emission at 548/48, using a polychroic beam splitter suitable for CFP/eYFP/mCherry. TAMRA images were captured with 1 sec exposure time, S2-eYFP with 5 sec exposure time, and GFP fusions with 0.5 to 1 sec exposure time. If stated in the figure legends, deconvolution was performed using SoftWorX 3.6.0 software (GE Healthcare Bio-Sciences, Pittsburgh, PA, USA).

### Fluorescence measurements using a plate reader

*L*. *lactis* was grown overnight in GM17 with appropriate antibiotics. Cells were collected by centrifugation, washed in sterile 1×PBS, inoculated 1:100 in GCDM* with 5 μg ml^-1^ chloramphenicol, and divided as quintuplos in wells of a transparent 96-wells microtitre plate. Fluorescence was measured with the following equipment and settings: Infinite F200 plate reader (Tecan Group Ltd., Männedorf, Switzerland) with I-control 1.10.4.0 software (Tecan Group Ltd.), GFP filter set (excitation at 485 nm and emission at 535 nm). GFP signals were collected as top readings with a gain setting of 60. GFP fluorescence was divided by the corresponding OD_600_ measurements to correct for cell density effects. The cultures were grown at 30°C to mid-exponential phase (equivalent to an OD_600_ of ~0.4) and induced with nisin A at a final concentration of 5 ng ml^-1^. *L*. *lactis* LG029(pNZ8048) was used as an empty vector control to obtain fold changes in expression of DnaK-GFP.

### Data analysis

MATLAB-based (Mathworks, Natick, MA, USA) MicrobeTracker (http://microbetracker.org) or Oufti (http://www.oufti.org) software were used to automatically track cell contours [67,68]. Both ImageJ (http://imagej.net/ImageJ) and MicrobeTracker software were used to detect fluorescent spots or measure single-cell fluorescence.

Spot detection was performed using a Gaussian fit algorithm available in the ImageJ plug-in ISBatch [18], collecting the coordinates of the spots relative to the image file as well as the Gaussian fitting parameters of each spot. Spots corresponding to intracellular signals were obtained using the tracked cell meshes, and those lying outside the meshes were discarded. The remaining spot coordinates were transformed and normalized to intracellular coordinates to fit in the dimension of one half of a coccoid model cell. To construct the model cell, the average *L*. *lactis* cell outline and nucleoid size were extracted from image files of live DAPI-stained *L*. *lacti*s NZ9000 cells using cell and nucleoid tracking (S1C Fig). The membrane-width inside the cell outline was estimated using images of cells expressing the fluorescent membrane protein BcaP-GFP. The resulting demi-coccoid form had a dimension of 800×900 nm, representing the average *width×0*.*5(length)* of an *L*. *lactis* cell grown in GCDM*. Remaining spots were conjointly projected into this model cell, and the resulting spot occupancy was plotted as density maps using MATLAB. The Gaussian fitting parameters obtained from the ISBatch plug-in were used to calculate the intensity (I) of fluorescent spots (I = *πw*^2^*h*, where *w* = full-width at half-maximum (FWHM); *h* = height or maximum of Gaussian fit parameter).

To obtain mRNA degradation curves, FISH was employed to obtain total TAMRA signals per cell for each time point after rifampicin addition as described in **Preparation of cells for *in vivo* fluorescence microscopy imaging or RNA FISH**. TAMRA signals minus background were extracted using MicrobeTracker. Bootstrapping (5×500 randomly picked cells per population) was performed to examine the accuracy of the sampling distribution. Single exponential decay curves described by *N*(*t*) = *N*_0_*e*^−*λt*^ were fitted to the averages of normalized median values obtained from independent experiments using MATLAB. Transcript half-lives were calculated from the decay coefficients (*λ*) using $t_{\frac{1}{2}}=\text{ ln}\left(2\right)/\lambda$.

The ObjectJ plug-in for ImageJ was employed to track cell lengths and S2-eYFP distribution along the Y-axes of the cells (https://sils.fnwi.uva.nl/bcb/objectj). All box-and-whisker plots (box plots) are represented according to the Tukey method for plotting whiskers.

To the determine the U-density of transcript groups representing cytoplasmic proteins or membrane proteins, the nucleotide sequences of genes encoding all cytoplasmic proteins and membrane proteins were identified and extracted from the genome sequences of *L*. *lactis* MG1363 and *E*. *coli* K12 using tools available at https://genome2d.molgenrug.nl. The number of thymines was divided by the total number of nucleotides of each gene to obtain the uracil density per gene. The codon usage indexes for both organism were obtained from http://genomes.urv.es/CAIcal.

## Supporting Information

S1 Fig Verification of protein production of 12bs-labeled transcripts and analysis of mRNA distribution inside fluorescent foci. **(A)** Analysis of overexpressed StrepII-tagged CodY (30.2 kDa) in cell-free extracts and of StrepII-tagged BcaP (50.6 kDa) in membrane fractions of *L*. *lactis* NZ9000 by Western hybridization using anti-StrepII antibodies. BcaP is known to migrate faster than expected on the basis of its molecular mass and typically forms a band at a position where proteins of around 37.5 kDa would migrate [13]. Control (-), empty-vector control. **(B)** Micrographs of *L*. *lactis* NZ9000 cells expressing *gfp*_*12bs*_ mRNA and GFP protein. PC = phase contrast. Scale bar represents 2 μm. **(C)** To obtain the model cell which served as a projection space used for the localization maps, we analyzed the area occupied by the nucleoid and the membrane inside of the obtained cell meshes. Using MicrobeTracker, we measured the length and width of the cell meshes (cell outline) as well as the nucleoids stained by DAPI (cyan) of 200 living cells in various states of division (left panel). Furthermore, we extracted the average thickness occupied by the membrane by analyzing living cells expressing the fluorescent membrane protein BcaP-GFP (left panel). For each of these parameters, a population average was obtained. Right panel depicts a schematic, scaled representation of a cell that had just divided, including the obtained length and width averages (displayed in μm). Black lines represent cell contour obtained from cell meshes, turquoise lines depict chromosomal area, and green lines depict cell membrane and cell wall. **(D)** The Gaussian fit parameters FWHM (full-width at half maximum of spot height (*h)*), and the spot intensity I (π(FWHM)2*h*) obtained from spot detection analyses were used to visualize the spatial distribution of transcripts within foci formed by *bcaP*_*12bs*_, *codY*_*12bs*_, *PS1Δ9*_*12bs*_, or *SUT1*_*12bs*_ mRNA. Heat maps were reconstructed from the FWHM values of each focus, all of which were plotted as a function of reciprocal magnitudes for all four transcripts. The white dashed lines serve as a reference and indicate the cut-off of maximal FWHM values reached of spots with increased FWHM-to-brightness ratios. Contrast bar: low (blue) to high (yellow) density of spot parameter distribution.
(TIF)

S2 Fig Localization of MS2-labeled transcripts. Overexpressed *codY*_*12bs*_, *bcaP*_*12bs*_, *PS1Δ9*_*12bs*_ or *SUT1*_*12bs*_ transcripts visualized *in vivo* with co-expressed MS2-GFP in *L*. *lactis* LG010 cells (left panels). Corresponding phase contrast pictures are shown in the center panels. Scale bar = 5 μm. Location maps of spot projections (right panels) from the MS2 datasets obtained from 794, 1290, 609, and 841 cells, respectively, highlighting the preferential localization of each overexpressed mRNA (Method described in Material and methods). Thick transparent lines: Cell boundaries including the portion occupied by cell wall and membrane as approximated using BcaP-GFP expressing cells (See S1C Fig). Thin transparent lines: Boundaries of chromosomal areas as approximated using DAPI staining in living cells (See S1C Fig). Scale bars depict the relative density of each mRNA species.
(TIF)

S3 Fig Co-localization of overexpressed membrane protein mRNA with cognate proteins or DnaK-GFP. **(A)** Deconvolved fluorescence micrographs of co-visualized *bcaP*_*12bs*_ mRNA and the corresponding membrane protein BcaP fused to GFP. The upper panels show cells in which BcaP-GFP aggregation seeds are present, while cells with dense polar *bcaP-gfp*_*12bs*_ mRNA clusters in combination with membrane-localized BcaP-GFP are exemplified in the lower panels. The yellow arrowhead in the right-upper panel indicates an overlapping fluorescent mRNA and protein focus. Right panels: false-colored overlays (red: mRNA; cyan: protein). **(B)** Fluorescence micrographs of *L*. *lactis* NZ9000 cells with TAMRA-labeled *PS1Δ9-gfp*_*12bs*_ transcripts (left panel), PS1Δ9-GFP (second panel from the left) and a deconvolved false-colored overlay (third panel from the left; red: *PS1Δ9-gfp*_*12bs*_; cyan: PS1Δ9-GFP). Right-most panel: Original false-colored overlay image. Yellow arrowheads indicate signals that show proximal localization of PS1Δ9-GFP protein and mRNA. **(C)** Upper two panels show fluorescence micrographs of DnaK-GFP localization in *L*. *lactis* LG029 cells collected and examined during exponential growth without stress. The upper left panel displays the effect on DnaK-GFP localization of fixation with 3.7% paraformaldehyde (PFA). The upper right panel shows the distribution of DnaK-GFP in living cells. The lower panels depict DnaK-GFP localization in *L*. *lactis* LG029 cells expressing either *bcaP*_*12bs*_ or *PS1Δ9*_*12bs*_. Cells were fixed with 3.7% PFA and prepared for FISH analysis prior to imaging. **(D)** Histograms and their fitted normal distributions of the distances between centers of PS1Δ9-GFP (grey) or DnaK-GFP (red) foci and polar *PS1Δ9-gfp*_*12bs*_ mRNA clusters in 100 single NZ9000 or LG029 cells, respectively. **(E)** Location maps of preferential localization of *PS1Δ9*_*12bs*_ mRNA, PS1Δ9-GFP, and DnaK-GFP. Intracellular coordinates of fluorescent foci corresponding to overexpressed *PS1Δ9*_*12bs*_ mRNA, and of co-visualized PS1Δ9-GFP or DnaK-GFP, reconstructed as described in **Data analysis**. Scale bars in all micrographs correspond to 2 μm.
(TIF)

S4 Fig Localization of pNZ-derived plasmids in single *L*. *lactis* cells. **(A)** Fluorescence micrographs of *L*. *lactis* LG045a cells constitutively expressing ParB-GFP, either carrying pNZ8048 without *parS* sequence (left panel) or pNZ8048 with consensus *parS* sequence (right panel). **(B)** pLG-BcaP plasmids visualized in *L*. *lactis* NZ9000 using DNA FISH and the TAMRA labeled *ms2* probes after non-induced cells were treated with RNase I to remove (complementary) RNA.
(TIF)

S5 FigEffect of disruption of translation of overexpressed transcripts on MS2-GFP localization and GFP production.**(A)** Fluorescence microscopy *L*. *lactis* LG010 cells expressing MS2-GFP, with or with Cm/Ery treatment. Treated cells display a partial exclusion of MS2-GFP from the nucleoid region. This is not the case in the non-treated cells. Scale bar is 2 μm. **(B)** The fluorescence of *L*. *lactis* NZ9000 cells overexpressing *gfp*_*12bs*_ transcripts with or without ribosomal binding sites (RBS). **(C)** Snap shots of time-lapse microscopy following the subcellular S2-eYFP distribution in *L*. *lactis*(*rpsB*::*rpsB-eYFP*) grown in nutrition-rich or nutrition-limiting conditions. Cells were grown to mid-exponential phase in GCDM*, after which samples were transferred to a microscope slide with an agarose patch either dissolved in GCDM* or 1×PBS supplemented with 0.5% glucose (GPBS). **(D)** The relative level of overexpressed *codY*_*12bs*_ (black), *bcaP*_*12bs*_ (red), *PS1Δ9*_*12bs*_ (grey), and *SUT1*_*12bs*_ (blue) transcripts as a function of time after rifampicin addition (at t = 0) was monitored using FISH. The data was fitted to single exponential decay, of which the curves are shown as solid lines. Error bars depict standard deviations obtained from bootstrapped datasets. **(E)** The dataset of [36] was used to determine the mRNA degradation rates of lactococcal transcripts. The log values of median half-lives, $\text{log}\left(t_{\frac{1}{2}}\right)$, of a total of 994, 787, and 996 transcripts at growth rate (*μ*) of 0.11, 0.51, and 0.80 *h*^−1^, respectively, were extracted and plotted as a function of the respective growth rates (open black circles). A best fit to the data (dashed black line) followed from $\text{log}\left(t_{\frac{1}{2}}\right)\;=\;-2.3\mu^{2}+\;0.6\mu +2.7$. The closed black circle indicates the median $\text{log}\left(t_{\frac{1}{2}}\right)$ at *μ* = 0.55*h*^−1^, corresponding to a median $t_{\frac{1}{2}}$ of 10.9 min. A similar strategy was employed to estimate the $t_{\frac{1}{2}}$ values of *codY* (open red circle) and *bcaP* (open blue circle) mRNA at *μ* = 0.55*h*^−1^, resulting in $\text{log}\left(t_{\frac{1}{2}}\;codY\right)\;=\;-2.0\mu^{2}+\;0.1\mu +2.4$ (dotted red line) and $\text{log}\left(t_{\frac{1}{2}}\;bcaP\right)\;=\;-5.2\mu^{2}+\;3.3\mu +2.2$ (dotted blue line). The lifetime of *codY*_*12bs*_ mRNA (t_1/2_ = 7.1 min) was in good agreement with that previously reported for *codY* mRNA (t_1/2_ = 7.4 min for μ = 0.55 h^-1^). This corresponds to a $t_{\frac{1}{2}}$ for *codY* or *bcap* mRNA of 6.4 min or 11.5 min, respectively, at *μ* = 0.55*h*^−1^. Breakdown of overexpressed *bcaP*_*12bs*_ mRNA ($t_{\frac{1}{2}}\;=\;6.2\text{ min}$) was at least not slower than that of endogenous *bcaP* mRNA. Note that media composition differs substantially between our measurements and those performed by Dressaire et al., possibly influencing specific mRNA lifetimes. **(F)** Degradation curves of *PS1Δ9*_*12bs*_ (grey squares) or *SUT1*_*12bs*_ (blue squares) of cells containing polar mRNA clusters (filled squares, solid lines) or cells without such foci (open squares, dashed lines). The data were fitted to single exponential decay functions, of which corresponding curves are depicted in the graph. **(G)** Degradation curves of *bcaP*_*12bs*_ or *PS1Δ9*_*12bs*_ transcripts with (red and grey dashed lines, respectively; replicated from **(D)**) or without functional RBS (red and grey solid lines).(TIF)

S1 Movie Time-lapse movie of *L*. *lactis* cells with MS2-GFP-labeled *bcaP*_*12bs*_ mRNA. (MOV)

S2 Movie Time-lapse movie of *L*. *lactis* cells with MS2-GFP-labeled *codY*_*12bs*_ mRNA. (MOV)

S3 Movie Time-lapse movie of *L*. *lactis* cells with MS2-GFP-labeled *PS1Δ9*_*12bs*_ mRNA. (MOV)

S4 Movie Time-lapse movie of *L*. *lactis* cells with MS2-GFP-labeled *SUT1*_*12bs*_ mRNA. (MOV)

S5 Movie Time-lapse movie of *L*. *lactis* cells with MS2-GFP-labeled *PS1Δ9*_*12bs*_ mRNA clusters that ceased to grow. Cells from a nisin-induced culture were transferred to a microscope slide with an agarose patch in growth medium without additional inducer. Scale bar represents 2 μm.
(AVI)

S6 Movie Time-lapse movie of *L*. *lactis* cells with MS2-GFP-labeled *PS1Δ9*_*12bs*_ mRNA clusters that were removed from the poles, after which the cells elongated without cell division. Cells from a nisin-induced culture were transferred to a microscope slide with an agarose patch in growth medium without additional inducer. Scale bar represents 2 μm.
(AVI)

S7 Movie Time-lapse movie of *L*. *lactis* cells with MS2-GFP-labeled *PS1Δ9*_*12bs*_ mRNA clusters that were removed from the poles, after which cell division was restored. Cells from a nisin-induced culture were transferred to a microscope slide with an agarose patch in growth medium without additional inducer nisin. Scale bar represents 2 μm.
(AVI)

S8 Movie Time-lapse movie of *L*. *lactis* cells in which MS2-GFP-labeled *bcaP*_*12bs*_ mRNA foci disappeared. Cells from a nisin-induced culture were transferred to a microscope slide with an agarose patch in growth medium without additional inducer. Scale bar represents 2 μm.
(AVI)

S1 Table Strains and plasmids used in this study. Ab^r^ = Antibiotic resistance. Amp = ampicillin, Cm = chloramphenicol, Ery = erythromycin.
(DOCX)

S2 Table Sequences of oligonucleotide used for plasmid and strain construction. Underlined nucleotides highlight restriction enzyme sites.
(DOCX)

S3 Table Sequences of oligonucleotide sequences used for FISH. (DOCX)

S1 Text Supplementary Results and Materials. Content: **1**. Validation of single-probe FISH for transcript visualization in *L*. *lactis*. **2**. Evaluation of the MS2 system for visualization of overproduced transcripts in living *L*. *lactis* cells. **3**. Plasmid and strain construction. **4**. S1 Text—Figures. **5**. S1 Text—References.
(DOCX)
