# Supplementary figures and images for: On the Spatial Organization of mRNA, Plasmids, and Ribosomes in a Bacterial Host Overexpressing Membrane Proteins
Source: PLoS Genet. 2016 Dec 15;12(12):e1006523. doi: 10.1371/journal.pgen.1006523 (PMC5201305; doi:10.1371/journal.pgen.1006523)

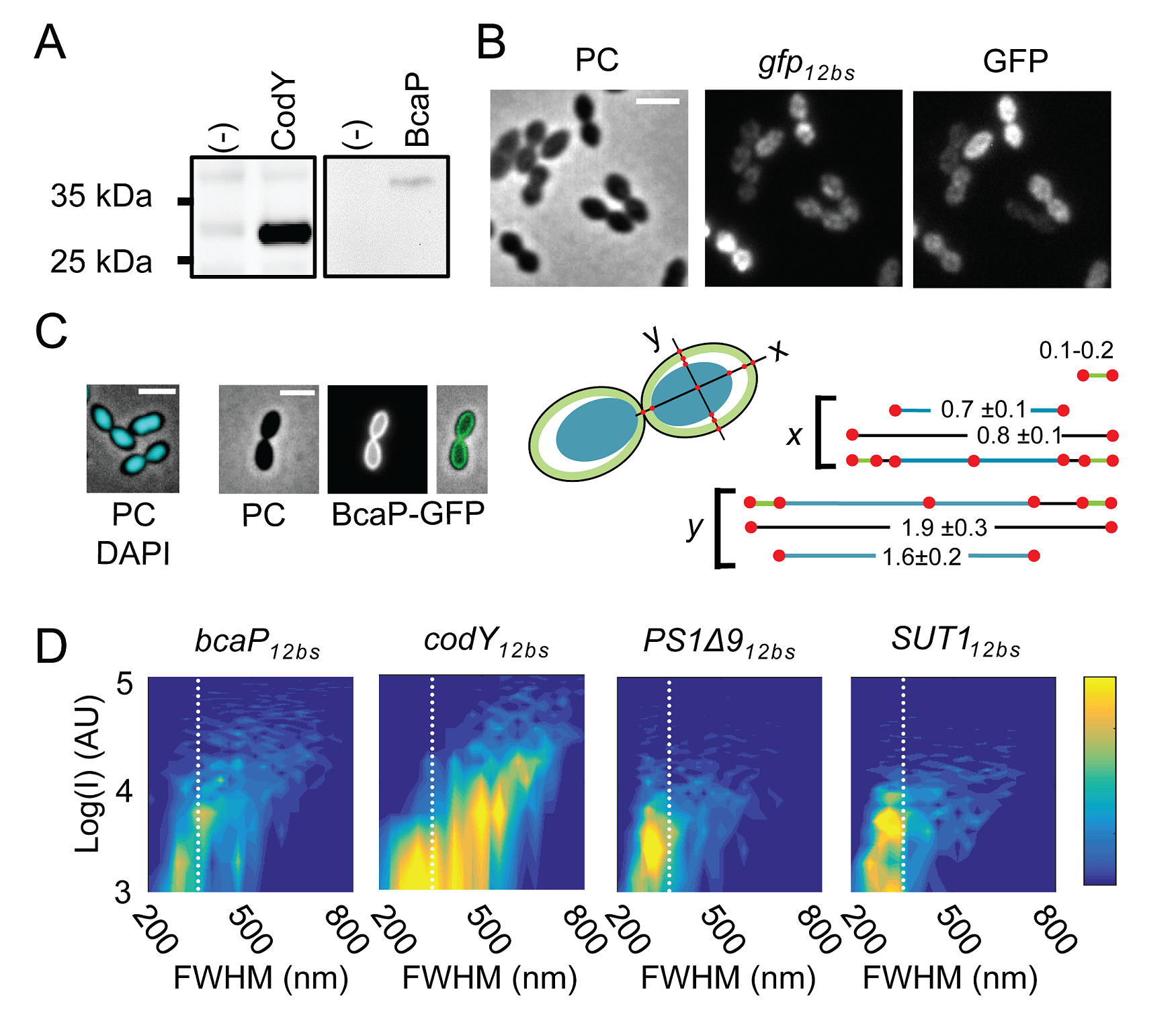

Supplement: S1 Fig — (A) Analysis of overexpressed StrepII-tagged CodY (30.2 kDa) in cell-free extracts and of StrepII-tagged BcaP (50.6 kDa) in membrane fractions of L. lactis NZ9000 by Western hybridization using anti-StrepII antibodies. BcaP is known to migrate faster than expected on the basis of its molecular mass and typically forms a band at a position where proteins of around 37.5 kDa would migrate [13]. Control (-), empty-vector control. (B) Micrographs of L. lactis NZ9000 cells expressing gfp12bs mRNA and GFP protein. PC = phase contrast. Scale bar represents 2 μm. (C) To obtain the model cell which served as a projection space used for the localization maps, we analyzed the area occupied by the nucleoid and the membrane inside of the obtained cell meshes. Using MicrobeTracker, we measured the length and width of the cell meshes (cell outline) as well as the nucleoids stained by DAPI (cyan) of 200 living cells in various states of division (left panel). Furthermore, we extracted the average thickness occupied by the membrane by analyzing living cells expressing the fluorescent membrane protein BcaP-GFP (left panel). For each of these parameters, a population average was obtained. Right panel depicts a schematic, scaled representation of a cell that had just divided, including the obtained length and width averages (displayed in μm). Black lines represent cell contour obtained from cell meshes, turquoise lines depict chromosomal area, and green lines depict cell membrane and cell wall. (D) The Gaussian fit parameters FWHM (full-width at half maximum of spot height (h)), and the spot intensity I (π(FWHM)2h) obtained from spot detection analyses were used to visualize the spatial distribution of transcripts within foci formed by bcaP12bs, codY12bs, PS1Δ912bs, or SUT112bs mRNA. Heat maps were reconstructed from the FWHM values of each focus, all of which were plotted as a function of reciprocal magnitudes for all four transcripts. The white dashed lines serve as a reference and in [file pgen.1006523.s001.tif]

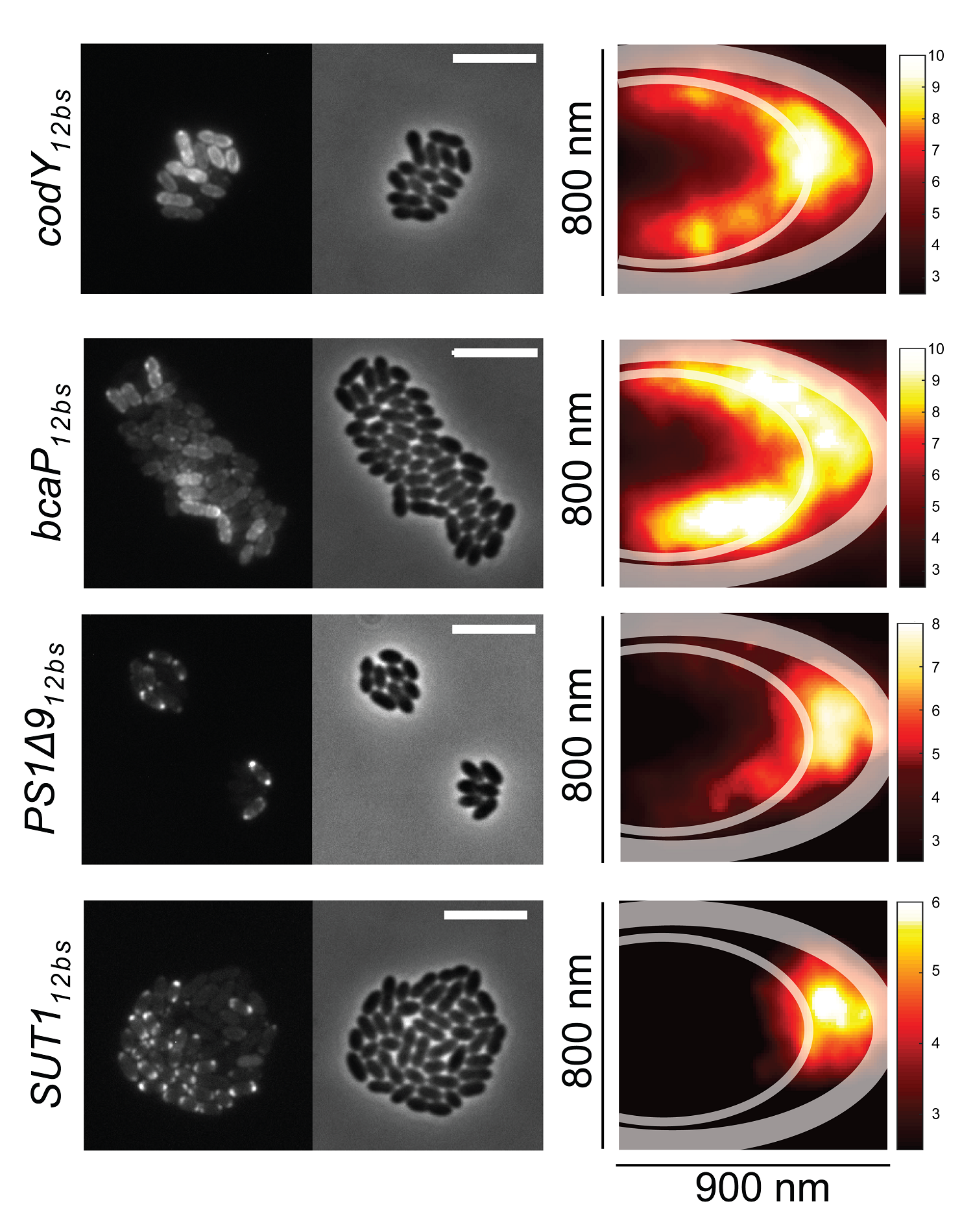

Supplement: S2 Fig — Overexpressed codY12bs, bcaP12bs, PS1Δ912bs or SUT112bs transcripts visualized in vivo with co-expressed MS2-GFP in L. lactis LG010 cells (left panels). Corresponding phase contrast pictures are shown in the center panels. Scale bar = 5 μm. Location maps of spot projections (right panels) from the MS2 datasets obtained from 794, 1290, 609, and 841 cells, respectively, highlighting the preferential localization of each overexpressed mRNA (Method described in Material and methods). Thick transparent lines: Cell boundaries including the portion occupied by cell wall and membrane as approximated using BcaP-GFP expressing cells (See S1C Fig). Thin transparent lines: Boundaries of chromosomal areas as approximated using DAPI staining in living cells (See S1C Fig). Scale bars depict the relative density of each mRNA species. (TIF) [file pgen.1006523.s002.tif]

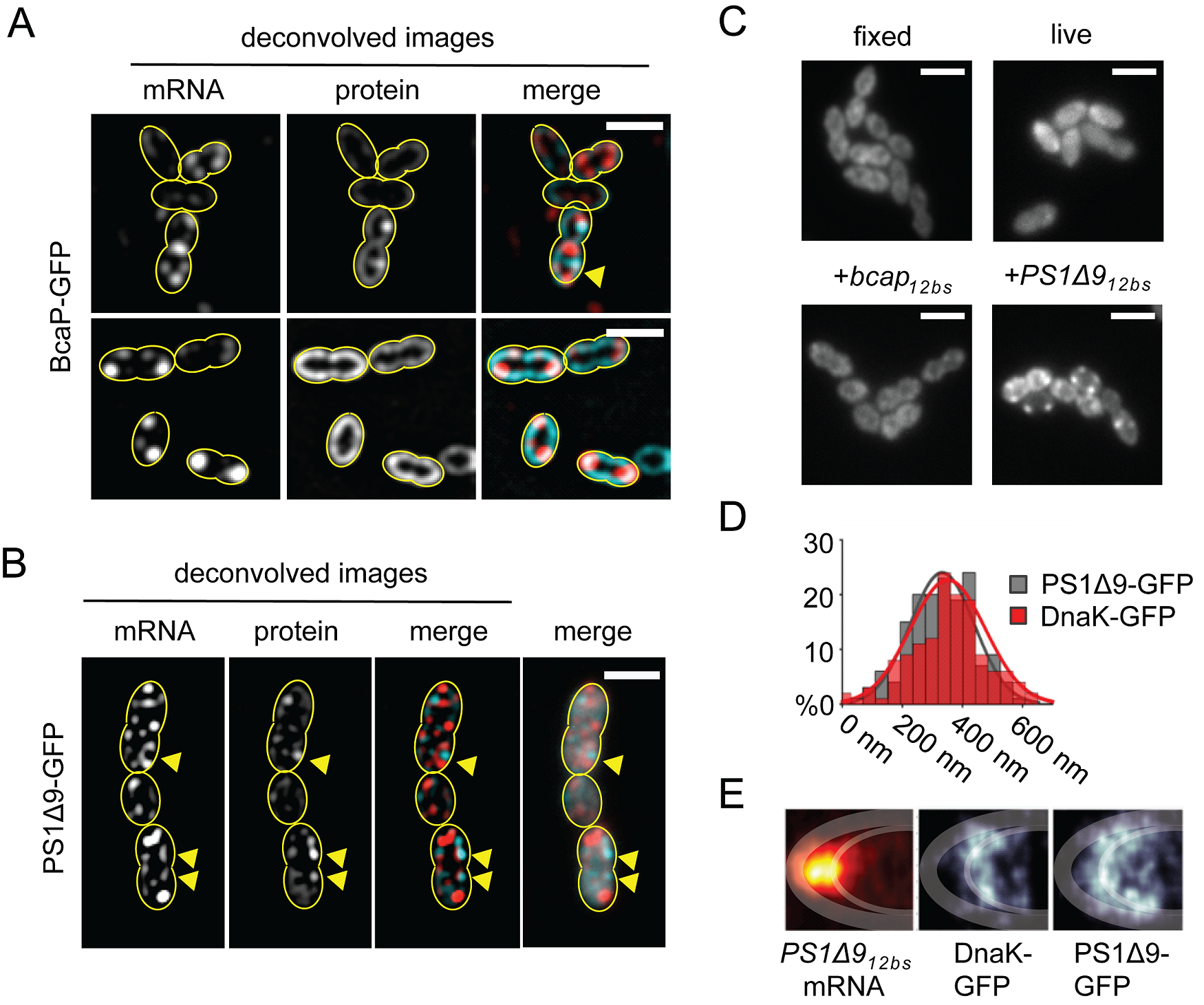

Supplement: S3 Fig — (A) Deconvolved fluorescence micrographs of co-visualized bcaP12bs mRNA and the corresponding membrane protein BcaP fused to GFP. The upper panels show cells in which BcaP-GFP aggregation seeds are present, while cells with dense polar bcaP-gfp12bs mRNA clusters in combination with membrane-localized BcaP-GFP are exemplified in the lower panels. The yellow arrowhead in the right-upper panel indicates an overlapping fluorescent mRNA and protein focus. Right panels: false-colored overlays (red: mRNA; cyan: protein). (B) Fluorescence micrographs of L. lactis NZ9000 cells with TAMRA-labeled PS1Δ9-gfp12bs transcripts (left panel), PS1Δ9-GFP (second panel from the left) and a deconvolved false-colored overlay (third panel from the left; red: PS1Δ9-gfp12bs; cyan: PS1Δ9-GFP). Right-most panel: Original false-colored overlay image. Yellow arrowheads indicate signals that show proximal localization of PS1Δ9-GFP protein and mRNA. (C) Upper two panels show fluorescence micrographs of DnaK-GFP localization in L. lactis LG029 cells collected and examined during exponential growth without stress. The upper left panel displays the effect on DnaK-GFP localization of fixation with 3.7% paraformaldehyde (PFA). The upper right panel shows the distribution of DnaK-GFP in living cells. The lower panels depict DnaK-GFP localization in L. lactis LG029 cells expressing either bcaP12bs or PS1Δ912bs. Cells were fixed with 3.7% PFA and prepared for FISH analysis prior to imaging. (D) Histograms and their fitted normal distributions of the distances between centers of PS1Δ9-GFP (grey) or DnaK-GFP (red) foci and polar PS1Δ9-gfp12bs mRNA clusters in 100 single NZ9000 or LG029 cells, respectively. (E) Location maps of preferential localization of PS1Δ912bs mRNA, PS1Δ9-GFP, and DnaK-GFP. Intracellular coordinates of fluorescent foci corresponding to overexpressed PS1Δ912bs mRNA, and of co-visualized PS1Δ9-GFP or DnaK-GFP, reconstructed as described in Data analysis. Scale bars in all micrographs co [file pgen.1006523.s003.tif]

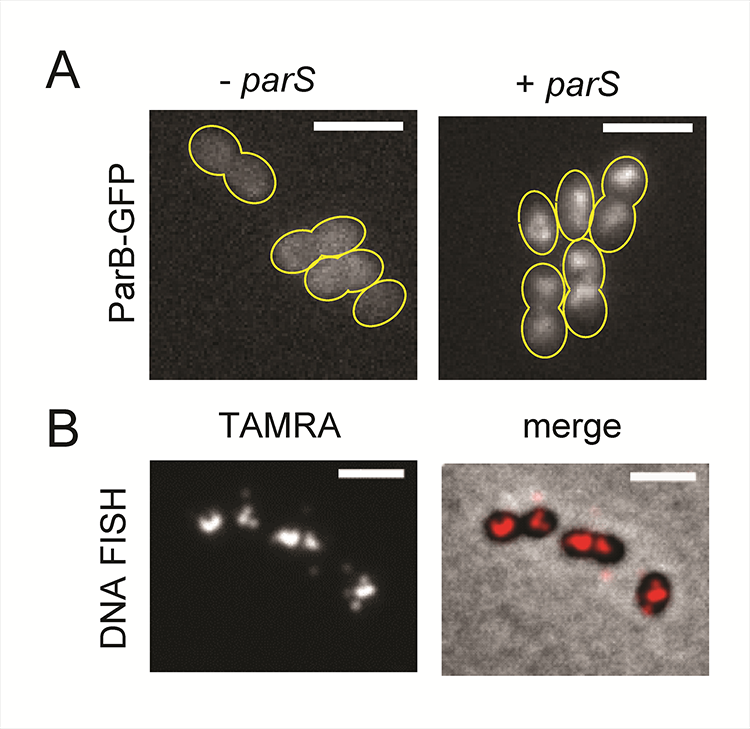

Supplement: S4 Fig — (A) Fluorescence micrographs of L. lactis LG045a cells constitutively expressing ParB-GFP, either carrying pNZ8048 without parS sequence (left panel) or pNZ8048 with consensus parS sequence (right panel). (B) pLG-BcaP plasmids visualized in L. lactis NZ9000 using DNA FISH and the TAMRA labeled ms2 probes after non-induced cells were treated with RNase I to remove (complementary) RNA. (TIF) [file pgen.1006523.s004.tif]

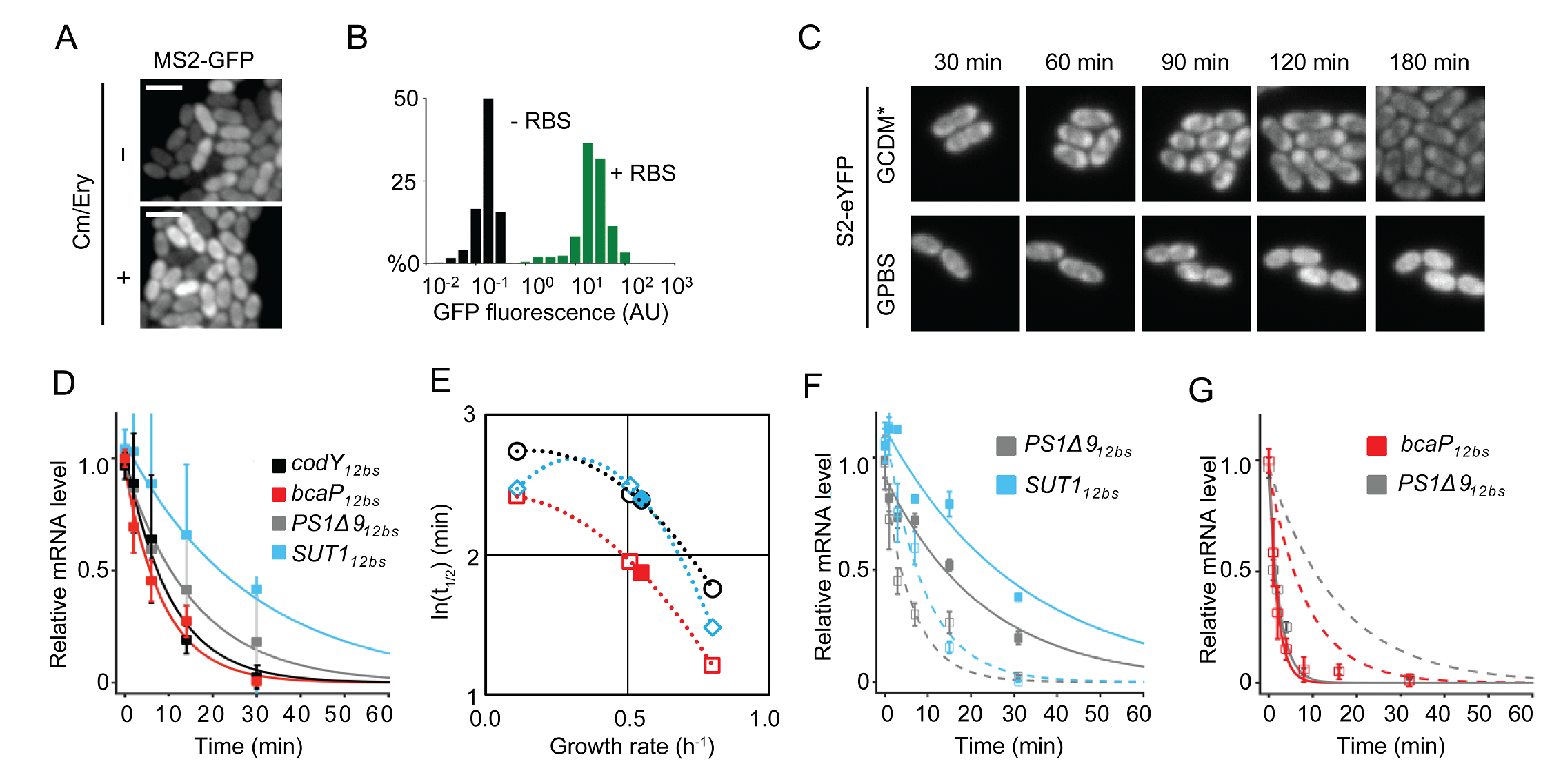

Supplement: S5 Fig — (A) Fluorescence microscopy L. lactis LG010 cells expressing MS2-GFP, with or with Cm/Ery treatment. Treated cells display a partial exclusion of MS2-GFP from the nucleoid region. This is not the case in the non-treated cells. Scale bar is 2 μm. (B) The fluorescence of L. lactis NZ9000 cells overexpressing gfp12bs transcripts with or without ribosomal binding sites (RBS). (C) Snap shots of time-lapse microscopy following the subcellular S2-eYFP distribution in L. lactis(rpsB::rpsB-eYFP) grown in nutrition-rich or nutrition-limiting conditions. Cells were grown to mid-exponential phase in GCDM*, after which samples were transferred to a microscope slide with an agarose patch either dissolved in GCDM* or 1×PBS supplemented with 0.5% glucose (GPBS). (D) The relative level of overexpressed codY12bs (black), bcaP12bs (red), PS1Δ912bs (grey), and SUT112bs (blue) transcripts as a function of time after rifampicin addition (at t = 0) was monitored using FISH. The data was fitted to single exponential decay, of which the curves are shown as solid lines. Error bars depict standard deviations obtained from bootstrapped datasets. (E) The dataset of [36] was used to determine the mRNA degradation rates of lactococcal transcripts. The log values of median half-lives, log(t12), of a total of 994, 787, and 996 transcripts at growth rate (μ) of 0.11, 0.51, and 0.80 h−1, respectively, were extracted and plotted as a function of the respective growth rates (open black circles). A best fit to the data (dashed black line) followed from log(t12) = −2.3μ2+ 0.6μ+2.7. The closed black circle indicates the median log(t12) at μ = 0.55h−1, corresponding to a median t12 of 10.9 min. A similar strategy was employed to estimate the t12 values of codY (open red circle) and bcaP (open blue circle) mRNA at μ = 0.55h−1, resulting in log(t12 codY) = −2.0μ2+ 0.1μ+2.4 (dotted red line) and log(t12 bcaP) = −5.2μ2+ 3.3μ+2.2 (dotted blue line). The lifetime of codY12bs mRNA (t1/2 = 7.1 min) was in good agr [file pgen.1006523.s005.tif]
