# Supplementary material for: On the Spatial Organization of mRNA, Plasmids, and Ribosomes in a Bacterial Host Overexpressing Membrane Proteins
Source: PLoS Genet. 2016 Dec 15;12(12):e1006523. doi: 10.1371/journal.pgen.1006523 (PMC5201305; doi:10.1371/journal.pgen.1006523)
Supplement: S1 Table — Abr = Antibiotic resistance. Amp = ampicillin, Cm = chloramphenicol, Ery = erythromycin. (DOCX) [file pgen.1006523.s014.docx]

| **strain** | **species** | **genetic marker(s)** | **Ab^r^** | **reference** |
| --- | --- | --- | --- | --- |
| NZ9000 | *L. lactis* | MG1363 *pepN::nisRK* |  | deRuyter 1995 |
| LG009 | *L. lactis* | NZ9000 *pseudo10::*P*nisA-MS2^ΔA^-sfgfp^m^* |  | this study |
| LG010 | *L. lactis* | NZ9000 *pseudo10::*P*nisA-MS2d^ΔA^-sfgfp^m^* |  | this study |
| LG029 | *L. lactis* | NZ9000 *dnaK::dnaK-sfgfp^m^* |  | this study |
| LG045a | *L. lactis* | NZ9000 *pseudo39::pSE39-*P*rnY-parB-sfgfp^m^* | Ery | this study |
| LG024a | *L. lactis* | NZ9000 *pseudo39::pSE39-*P*rnY-rnY-sfgfp^m^* | Ery | this study |
| NZ9000(*rpsB::rpsB-eyfp*) | *L. lactis* | NZ9000 *rpsB::rpsB-eyfp* |  | this study; Poolman lab |
| DH5α | *E. coli* | *fhuA2 lac(del)U169 phoA glnV44 Φ80' lacZ(del)M15 gyrA96 recA1 relA1 endA1 thi-1 hsdR17* |  | Taylor, RG et al. (1993) |
| **plasmid name** | **host** | **description** | **Ab^r^** | **reference** |
| pNZ8048 | *L. lactis* | NICE system | Cm | PMID: 8837421 |
| pSEUDO | *E. coli* | *L. lactis* integration vector | Ery | Pinto et al. AEM. 2011 |
| pCS1966 | *E. coli* | *L. lactis* integration vector | Ery | Solem et al. AEM. 2008 |
| pSEUDO39:*mKate2* | *E. coli* | *L. lactis* integration vector | Ery | Veening/Kuipers/Kok lab |
| pSEUDO39:*parB-mKate2* | *E. coli* | *L. lactis* integration vector | Ery | Veening/Kuipers/Kok lab |
| pSEUDO::P*usp45-sfgfp(Bs)* | *E. coli* | *L. lactis* integration vector | Ery | Overkamp et al. AEM. 2013 |
| pSEUDO10:*mg3* | *E. coli* | *L. lactis* integration vector; for nisin-controlled expression of MS2^ΔA^-sfGFP^m^ | Ery | this study |
| pSEUDO10:*mg4* | *E. coli* | *L. lactis* integration vector; for nisin-controlled expression of MS2d^ΔA^-sfGFP^m^ | Ery | this study |
| pSEUDO39:P*rnY-parB-gfp* | *E. coli* | *L. lactis* integration vector for insertion of *parB*-sfgfp^m^* under control of the *L. lactis* P*rnY* | Ery | this study |
| pCS1966-*dnaK-gfp* | *E. coli* | *L. lactis* integration vector for replacement of *dnaK* with *dnaK-sfgfp^m^* | Ery | this study |
| pLG-BcaP | *L. lactis* | for nisin-controlled expression of *bcaP(strepII)_12bs_* | Cm | this study |
| pLG-BcaP-GFP | *L. lactis* | for nisin-controlled expression of *bcaP-gfp_12bs_* | Cm | this study |
| pNZ-BcaP-H6 | *L. lactis* | for nisin-controlled expression of BcaP-H6 | Cm | Pinto et al. PlosOne 2011 |
| pLG-CodY | *L. lactis* | for nisin-controlled expression of *codY(strepII)_12bs_* | Cm | this study |
| pLG-PS1Δ9 | *L. lactis* | for nisin-controlled expression of *PS1Δ9 (strepII)_12bs_* | Cm | this study |
| pLG-PS1Δ9-GFP | *L. lactis* | for nisin-controlled expression of *PS1Δ9 -gfp_12bs_* | Cm | this study |
| pNZ-PS1Δ9 | *L. lactis* | for nisin-controlled expression of H10-PS1Δ9 of *Homo sapiens* | Cm | Marreddy et al. PlosOne 2011 |
| pLG-SUT1 | *L. lactis* | for nisin-controlled expression of *SUT1(strepII)_12bs_* | Cm | this study |
| pNZ-StSUT1 | *L. lactis* | for nisin-controlled expression of H10-SUT1 of *Solanum tuberosum* | Cm | Marreddy et al. PlosOne 2011 |
| pLG-GFP | *L. lactis* | for nisin-controlled expression of *gfp_12bs_* | Cm | this study |
| pLG-2tmBcaP-PS1Δ9 | *L. lactis* | for nisin-controlled expression of *PS1Δ9^N^(strepII)_12bs_ w*hich has its first 2 TMDs replaced by those of BcaP | Cm | this study |
| pLG-2tmPS1-BcaP | *L. lactis* | for nisin-controlled expression of *BcaP^N^(strepII)_12bs_ w*hich has its first 2 TMDs replaced by those of PS1Δ9 | Cm | this study |
| pLG-BLS-PS1Δ9 | *L. lactis* | for nisin-controlled expression of *BLS-PS1Δ9(strepII)_12bs_ w*hich has the first 2 TMDs of BcaP, followed by a TEV protease site and the complete PS1Δ9 sequence. | Cm | this study |
| pNZ8048(*parS*) | *L. lactis* | contains ParB-binding sequence *parS* | Cm | this study |
| pLG(*parS*)-BcaP | *L. lactis* | for nisin-controlled expression of *bcaP(strepII)_12bs_*; contains ParB-binding sequence *parS* | Cm | this study |
| pLG(*parS*)-PS1Δ9 | *L. lactis* | for nisin-controlled expression of *PS1Δ9 (strepII)_12bs_*; contains ParB-binding sequence *parS* | Cm | this study |
| pLG_ΔRBS_-BcaP | *L. lactis* | for nisin-controlled expression of *RBS-less bcaP(strepII)_12bs_* | Cm | this study |
| pLG_ΔRBS_-PS1Δ9 | *L. lactis* | for nisin-controlled expression of *RBS-less PS1Δ9 (strepII)_12bs_* | Cm | this study |
| pLG_ΔRBS_-GFP | *L. lactis* | for nisin-controlled expression of *RBS-less gfp_12bs_* | Cm | this study |
| pLG-01 | *L. lactis* | *sfgfp(Bs)* N-terminally extended with polylinker and inserted in pNZ8048 via PstI and SpeI | Cm | this study |
| pLG-01m | *L. lactis* | Monomeric version of *sfgfp(Bs)* N-terminally extended with polylinker and inserted in pNZ8048 via PstI and SpeI | Cm | this study |
| pLG-MG1 | *L. lactis* | for nisin-controlled expression of MS2^wt^-sfGFP^m^ | Cm | this study |
| pLG-MG2 | *L. lactis* | for nisin-controlled expression of MS2d^wt^-sfGFP^m^ | Cm | this study |
| pLG-MG3 | *L. lactis* | for nisin-controlled expression of MS2^ΔA^-sfGFP^m^ | Cm | this study |
| pLG-MG4 | *L. lactis* | for nisin-controlled expression of MS2d^ΔA^-sfGFP^m^ | Cm | this study |
| pZA25GFP | *E. coli* | contains gene encoding full-length MS2 phage coat protein, MS2^wt^ | Amp | Nevo-Dinur et al. Science. 2011 |
| pMS2-GFP | *E. coli* | contains gene encoding aggregation-deficient MS2 phage coat protein, MS2^ΔA^ | Amp | Fusco et al. Curr Biol. 2003 |
| pSL-MS2-12x | *E. coli* | contains 12 MS2 binding sites | Amp | Bertrand et al. Mol Cell. 1998 |
| pSL-bcaP-MS2-12x | *E. coli* | pSL-MS2-12x with *bcaP* inserted upstream of 12 MS2 binding sites | Amp | this study |
| pSL-PS1-MS2-12x | *E. coli* | pSL-MS2-12x with *PS1* inserted upstream of 12 MS2 binding sites | Amp | this study |

**Table S1. Strains and plasmids used in this study.** Ab^r^=Antibiotic resistance. Amp = ampicillin, Cm = chloramphenicol, Ery = erythromycin.
