# Supplementary material for: On the Spatial Organization of mRNA, Plasmids, and Ribosomes in a Bacterial Host Overexpressing Membrane Proteins
Source: PLoS Genet. 2016 Dec 15;12(12):e1006523. doi: 10.1371/journal.pgen.1006523 (PMC5201305; doi:10.1371/journal.pgen.1006523)
Supplement: S2 Table — Underlined nucleotides highlight restriction enzyme sites. (DOCX) [file pgen.1006523.s015.docx]

| **Primer name** | **Sequence (5' → 3')** |
| --- | --- |
| MS2-FB-NcoI | GCGCCATGGCTTCTAACTTTACTCAG |
| MS2-RB-PstI | CGCTGCAGGTAGATGCCGGAGTTTGCTGCGA |
| MS2D-F-NheI | CTGCTAGCATGGCTTCTAACTTTACTCAGTTCG |
| MS2D-R-NheI | GCGCTAGCGTAGATGCCGGAGTTTGCTGC |
| pBvdZ001 | CGCCCCGGGCCCCGTTAGTTGAAGAAGG |
| pBvdZ002 | GCGGGATCCGTCCTTTAATTGGTGGACAAATTTA |
| pJS89 | GGGGGCTAAATTTAGCCCCCTTTTCTAATGTCACTAACCTGCCCCG |
| pJS90 | GGGGGCTAAATTTAGCCCCCCCGACTGTAAAAAGTACAGTCGGC |
| pLVG017b | CGCCATGGGATTTATGAGAAAAGCCG |
| pLVG018b | CGGGATCCTTATCATTTTTCAAATTGAGGATGTGACCAAGCAGAATTCTTCTTTTTGCGACGATTTCC |
| pLVG019b | CGCCATGGGTGGTGGATTTGCTACAG |
| pLVG020b | CGGGATCCTTATCATTTTTCAAATTGAGGATGTGACCAAGAATTCCCTTGGAAGTATAAATTTTCG |
| pLVG028b | CGCCATGGCTACATTACTTGAAAAAAC |
| pLVG029b | CGGGATCCTTATCATTTTTCAAATTGAGGATGTGACCAAGAATTCAAATTACGTCCAGCAAG |
| pLVG042A | CAACTCAAAGCAAATTATCAAAAGACCCAAATGAAAAGCG |
| pLVG043A | GGGTCTTTTGATAATTTGCTTTGAGTTGATAAATAATGG |
| pLVG052b | ATTCCCCGGGCAUGCGACAGTTATTGAAGTAGCTG |
| pLVG060 | AGCGGGATCTGGUGGAGAAGCTGAAG |
| pLVG061 | ACCTTGACTATUATTACTTATAAAGCTCATCCATGC |
| pLVG062 | AATAGTCAAGGUCGGCAATTCTGC |
| pLVG063 | ATGCCCGGGGAAUTCAGACTGGAAGC |
| pLVG071 | ATGGTGAGTGCCUCCTTATAATTT |
| pLVG072 | AGTGAGGGTTAAUTGCGCGCTTG |
| pLVG073 | ATGTTACTGCUGATAATGTAGATATC |
| pLVG096 | ACCAGATCCCGCUTTCTTTTTGCGACGATTTCC |
| pLVG106 | ATGCTTGGTCACAUCCTCAAT |
| pLVG107 | AGGCACTCACCAUGGGTGAGAATTTATATTTTCAAGGTG |
| pLVG108 | ATGTGACCAAGCAUGTTTAATAGAGAGCCCCATGG |
| pLVG109 | ATAAATTATAAUTCGGCACTCACCATGG |
| pLVG110 | ATTATAATTTAUTTTGTAGTTCCTTCGAAC |
| pLVG117 | ACCAGATCCCGCUTTCATCAACAACTCAATCAATTTTTTC |
| pLVG118 | AGAGGAGACTAAUGAGTAATAGTTTCGGATTTACAG |
| pLVG119 | ATTAGTCTCCTCUAAAGTGAGTAATCATTCC |
| pLVG120 | ATGTGACCACTTUTAAAGCTCATCCATGCC |
| pLVG121 | TAAGTGGTCACAUCCTCAATTTGAAAAATG |
| pLVG122 | AGGCACTCACCAUGGCAAAAGGAGAAGAGCTG |
| pLVG139B | AGTGCCTCCTUATAATTTATTTTGTAGTTCCTTCGAACG |
| pLVG140 | AAGGAGGCACUCACCATGGG |
| pLVG141 | ACATTTCCCTGUATTTATACAGAACCACC |
| pLVG142B | ACAGGGAAATGUCAACAGTAATGCCCTTTGC |
| pLVG143 | ATAGCAAGCAUAAGCCAAGGCCAC |
| pLVG144B | ATGCTTGCTAUAAGGTCATCCATGCCTGG |
| pLVG145 | ACCAGATCCCGCUTGCCCTTGGAAGTATAAATTTTC |
| pLVG146 | AAATTCTCCTCUCCACCAGATCCAGCATAAGCCAAGGCCAC |
| pLVG149 | AGAGGAGAATTUGTATTTTCAGGGTGGTGGATTTGCTACAGAG |
| pLVG163 | AGCAGTAACAUTTGACGAATTGACACATGATC |
| pLVG164 | ACCAGATCCCGCUTTGCTTTCTTCAAAGTCGCCATC |
| pLVG165 | AATAGTCAAGGUTATTTTAAAAAAGTACTGATCGTTTCGTC |
| pLVG166 | ATTAACCCTCACUCAAAGGGAAGAGTACAAATCGTG |
| pTE186 | GGCCGCTGCAGGCGGGATCTGGTGGAGAAGCTGAAGCTAAAGGATTGAAAGGAGAAGAGCTGTTCACAGG |
| pTE187 | CGACCTTGACTAGTGCTCATTATTACTTATAAAGCTCATCCATGC |

**Table S2. Sequences of oligonucleotide used for plasmid and strain construction.** Underlined nucleotides highlight restriction enzyme sites.
