# Supplementary material for: On the Spatial Organization of mRNA, Plasmids, and Ribosomes in a Bacterial Host Overexpressing Membrane Proteins
Source: PLoS Genet. 2016 Dec 15;12(12):e1006523. doi: 10.1371/journal.pgen.1006523 (PMC5201305; doi:10.1371/journal.pgen.1006523)
Supplement: S3 Table — (DOCX) [file pgen.1006523.s016.docx]

| **Probe name** | **Sequence (5' → 3')** |
| --- | --- |
| MS2-aptamer FISH probe | TAMRA-5'-CTGCAGACATGGGTGATCCTC-3'-TAMRA |
| bcaP_probe4 | TAMRA-5'-GCCAACAAAGGCAACAGCAA-3'-TAMRA |
| bcaP_probe5 | TAMRA-5'-CACCATCTGTACCAAAAGGG-3'-TAMRA |
| bcaP_probe6 | TAMRA-5'-GCCGAGAGCAAAATGACCAA-3'-TAMRA |
| PS1_probe4 | TAMRA-5'-GTCGTCCATTAGATAATGGC-3'-TAMRA |
| PS1_probe5 | TAMRA-5'-TATTTCAATGTCAGCTCCTC-3'-TAMRA |
| PS1_probe6 | TAMRA-5'-TGACTTAATGGTAGCCACGA-3'-TAMRA |

**Table S3. Sequences of oligonucleotide sequences used for FISH.**
