# Supplementary material for: On the Spatial Organization of mRNA, Plasmids, and Ribosomes in a Bacterial Host Overexpressing Membrane Proteins
Source: PLoS Genet. 2016 Dec 15;12(12):e1006523. doi: 10.1371/journal.pgen.1006523 (PMC5201305; doi:10.1371/journal.pgen.1006523)
Supplement: S1 Text — Content: 1. Validation of single-probe FISH for transcript visualization in L. lactis. 2. Evaluation of the MS2 system for visualization of overproduced transcripts in living L. lactis cells. 3. Plasmid and strain construction. 4. S1 Text—Figures. 5. S1 Text—References. (DOCX) [file pgen.1006523.s017.docx]

**S1 Text – Supplementary Results and Materials**

**On the spatial organization of mRNA, plasmids, and ribosomes in a bacterial host overexpressing membrane proteins**

Lieke A. van Gijtenbeek^1^, Andrew Robinson^#2^, Antoine M. van Oijen^#2^, Bert Poolman^2,3^, and Jan Kok*^1^

**Content**

1. **Validation of single-probe FISH for transcript visualization in *L. lactis***
2. **Evaluation of the MS2 system for visualization of overproduced transcripts in living *L. lactis* cells**
3. **Plasmid and strain construction**
4. **S1 Text - Figures**
5. **S1 Text - References**
6. **Validation of single-probe FISH for transcript visualization in *L. lactis***

To determine localization and quantity upon overexpression of specific mRNA molecules in *L. lactis*, we first applied single probe (sp) FISH as essentially described in [1]. To avoid high costs in probe design and to make mRNA labeling compatible for both FISH *and* MS2 recognition, an array of 12 MS2 binding sequences (abbreviated to *12bs*) was introduced between the stop codon and the transcriptional terminator of the transcripts under study. The *12bs*-array served as a target for multiple hybridizations to a 20-nts oligonucleotide *ms2* probe labeled at both the 5’ and 3’ ends with carboxytetramethylrhodamine (TAMRA). The distance of 20-nts should be enough to avoid quenching of the adjacent dyes [2]. 3’-UTR mRNA-tagging was employed for two reasons: It does not interfere with translation of the preceding gene-coding region on the mRNA, and only fully transcribed mRNA molecules are visualized (**S1 Text - Fig 1A**).

To investigate spFISH functionality in *L. lactis*, chimeric transcripts containing the *bcaP* or the *PS1Δ9* gene, each extended with a *12bs*-array, were placed under the control of the nisin-inducible *P_nisA_* promoter on plasmid pNZ8048 and introduced in *L. lactis* NZ9000 [3]. The resulting strains were grown in rich chemically defined medium (GCDM*), until they had reached a density corresponding to an OD_600_ of 0.4, at which point the cultures were split in two. Half of each culture was induced with 5 ng ml^-1^ nisin A to start the expression of the transcripts, while the remaining, uninduced, cultures served as negative controls. Following another hour of incubation, the cells were collected and applied to the FISH protocol as set forth by Skinner and co-workers using the *ms2* probe [1,4]. On average, a 60-fold increase in TAMRA-signal was obtained in induced cells compared to the negative control cells (**S1 Text - Fig 1B**). Furthermore, distinct mRNA localization patterns were observed between *bcaP_12bs_* and *PS1Δ9_12bs_* (**S1 Text - Fig 1C**).

To study possible adverse effects of the *12bs*-extension on the mRNA molecules, FISH probes targeting the protein-coding regions of *bcaP* and *PS1Δ9* mRNA were designed (**S1 Text - Fig 1A**). Three dually-labeled oligonucleotides for each mRNA sufficed to visualize the overexpressed transcripts (**S1 Text - Fig 1D**). The use of gene-specific probes yielded similar localization patterns for *bcaP* or *PS1Δ9* mRNA with or without the *12bs*-array (**S1 Text - Fig 1D**). Moreover, the observed patterns were similar to those of cells expressing *bcaP_12bs_* or *PS1Δ9_12bs_* and that were stained with the site-specific *ms2* probe. Hence, the localization patterns of each type of transcript appeared to be (1) independent of the presence of the *12bs*-array at their 3’ ends and (2) similar whether obtained with the *ms2* or the gene-specific probes. MS2-tagging is known to impede degradation of transcripts due to the attachment of multiple RNA hairpin repeats to which MS2 proteins are bound. However, it is not clear whether the RNA extension alone, without bound MS2 proteins, has any significant effect on mRNA breakdown. We examined the degradation rate after rifampicin addition of overexpressed *bcaP* and *PS1Δ9* transcripts with or without *12bs*-array using FISH probes targeting either the gene-coding region or the *12bs*-array. The probe-mRNA combinations are depicted in **S1 Text - Fig 1A**. Specific decay curves for each transcript were obtained using the gene-specific probes; these were independent of the presence of the *12bs*-appendix (**S1 Text - Fig 1E)**. In addition, the half-lives obtained with the *ms2* and gene-specific probes for each type of mRNA are similar (**S1 Text - Fig 1E**). The degradation curves of *bcaP* or *PS1Δ9* acquired with the different probes were combined (**S1 Text - Fig 1F**). The results show that the variation in decay rates of the same transcript monitored with different probes was smaller than that between *bcaP* and *PS1Δ9* mRNA. Together, these data indicate that the *12bs*-array alone does not influence the specific localization adopted by *bcaP* and *PS1Δ9* mRNA, nor does it significantly affect transcript degradation. The introduction of the *12bs*-array to mRNA molecules of interest, and its subsequent use as a target sequence for spFISH, is a valuable method to achieve information on the characteristics of overexpressed mRNA molecules, albeit that the data is static.

1. **Evaluation of the MS2 system for visualization of overproduced transcripts in living *L. lactis* cells**

The MS2 phage coat protein (MS2) and its RNA binding sequence have been used to visualize transcripts in living *E. coli, Caulobacter crescentus* and *B. subtilis* cells [5–9]. MS2 recognizes its target RNA sequence as a dimer [10,11]. The construction of a single-chain tandem dimer of MS2 enhanced binding to reporter mRNA [12]. As full-length MS2 is prone to aggregation above certain concentration, an aggregation-deficient MS2 was developed; it lacks the domain required for self-assembly [5,13]. However, aggregation-deficient MS2 has reduced RNA binding capacity [6]. To set up the MS2 system in *L. lactis*, we therefore first examined the expression and localization of various MS2s in this organism. We fused the genes of the various MS2 variants to that of a monomeric version of a superfolder GFP (sfGFP^m^; V206K substitution) that optimally performs in *L. lactis* (sfGFP(bs)) [14]. The two proteins in a chimera were connected via a flexible peptide linker (AGSGGEAEA). Besides the aggregation-prone full-length MS2 protein, its dimerized version (MS2d), and the assembly-deficient MS2 protein (MS2 dIFG), we constructed a tandem dimer of the aggregation-deficient MS2 variant (MS2d dIFG; **S1 Text - Fig 2A**). The latter incorporates both the benefits of reduced aggregation and enhanced binding to its target RNA. The respective *ms2-gfp* genes were placed under control of P*_nisA_* on plasmid pNZ8048 and introduced in *L. lactis* NZ9000. To examine basal MS2-GFP expression levels and self-assembly propensities, each MS2-GFP protein (designated MG1 to MG4; **S1 Text - Fig 2A**) was overexpressed for 1 hr by the addition of 5 ng ml^-1^ of nisin to cells in mid-exponential phase. Single cells expressing the MS2 tandem dimer (MG2 or MG4), analyzed using fluorescence microscopy, possessed enhanced fluorescent signals compared to those expressing monomeric MS2 variants (**S1 Text - Fig 2B**). Fluorescence microscopy revealed that extended (>1 hr) and high (>5 ng ml^-1^ nisin) expression resulted in aggregation of full-length but not of aggregation-deficient MS2s, which made us discard the use of MG1 and MG2 (**S1 Text - Fig 2C**). Although cells expressing MG3 show reduced fluorescence compared to MG4, twice the number of monomeric MG3 can bind compared to the dimeric MS2 variants, leading to, in theory, an increased signal-to-noise ratio. As mentioned above, a tandem repeat of MS2 adopts its dimeric structure at higher rates, and therefore was found to have a more stable and enhanced binding affinity [12]. Hence, MG3 and MG4 were selected for further realization of the MS2 system in *L. lactis*.

The nisin-inducible P*_nisA_* promoter was employed for expression of both MS2-GFP protein and target RNA. The major pitfall of this approach is that expression of both elements cannot be modulated separately. On the other hand, we reasoned that the intracellular expression ratio between MS2-GFP and target RNA would be more or less constant when using the same inducible promoter. This is illustrated by single-cell measurements of eYFP and mKate2 co-expression from pIL253 or pNZ8048, respectively, of which the genes were both placed under the control of P*_nisA_* (**S1 Text - Fig 2D**). This is especially useful when the level of expression is heterogeneous, which is a characteristic of many inducible promoters, including P*_nisA_* [15]. Initially, both elements were cloned downstream of two separate *nisA* promoters located on a pNZ8048-derived duet gene expression vector (P2nis; kindly provided by A. Steen). Unfortunately, dual gene expression led to an imbalance of available MS2 binding sequences and MS2-GFP proteins after certain induction levels were reached. This resulted in aggregate-formation as well as high background fluorescence caused by a surplus of unbound MS2-GFP molecules. Therefore, a DNA fragment containing the genes of MG3 or MG4 downstream of P*_nisA_* and followed by a transcriptional terminator, was introduced in single-copy at the *pseudo10* locus on the chromosome of *L. lactis* NZ9000 [16], creating strain LG009 and LG010, respectively.

Depending on the purpose of previous studies, between 6 and 96 MS2 binding sites were introduced either on the 5’- or 3’-end of transcripts. We chose to introduce arrays of 6 or 12 MS2 binding sequences (abbreviated to *6bs* or *12bs*) immediately downstream of the stop codon at the 3’ UTR of the transcripts, in order to solely visualize fully transcribed mRNA molecules (**S1 Text - Fig 2E**). As we examine here a (membrane) protein overexpression system, we anticipated that one of these short arrays would suffice to obtain a good signal-to-noise ratio. Determination of the net fluorescence after 1 hr of induction with 5 ng ml^-1^ nisin revealed that the chromosomal location of the MS2-GFP encoding genes led to an order of magnitude reduction in fluorescence per cell compared to expression from pNZ8048 (**S1 Text - Fig 2F**). A pNZ8048 vector containing the *L. lactis* *codY* gene extended with the *6bs*- or *12bs*-array was introduced in LG009 and LG010, after which fluorescent patterns were examined upon similar induction conditions. Results of the different combinations are shown in **S1 Text - Fig 2G**. From the data, we conclude that the highest signal-to-noise ratio was obtained using strain LG010 in combination with a gene carrying a *12bs*-array.

Localization patterns of *bcaP_12bs_* mRNA obtained with the MS2 system in strain LG010 were certified with TAMRA-labeled FISH probes targeting the MS2-binding sites. TAMRA signals co-localized with MG4, although a correlation between GFP and TAMRA levels was absent (**S1 Text - Fig 2H**). Binding of MG4 proteins to the hairpin loops in the MS2 binding region could prevent hybridization of the FISH probes, but the fact that we observe a TAMRA signal indicated that not all MS2 binding sites are occupied by MG4, as has been reported before [12,17]. Although the results validate the use of MS2 to study *in vivo* localization and dynamics of overexpressed transcripts, we realize that this method is quite invasive and could increase transcript lifetime. To investigate the effect of MS2-GFP binding on *bcaP_12bs_* mRNA degradation, cells expressing *bcaP_12bs_* mRNA in the presence or absence of MG4 were treated with rifampicin, after which *bcaP_12bs_* degradation curves were obtained using FISH (**S1 Text - Fig 2I**). In both cases, a half-life of 6.6 min was measured, while co-localization of MG4 with TAMRA probes remained after extended rifampicin treatment (**S1 Text - Fig 2J**). As expected, fluorescent signals of MG4 did not diminish after addition of rifampicin, but mainly increased due to additional GFP maturation (data not shown).

Finally, the spatiotemporal behavior of *bcaP_12bs_* mRNA bound by MG4 was examined in *L. lactis* LG010*.* Expression of target RNA and MS2-GFP was induced during 1 hr by the addition of nisin to a final concentration of 5 ng ml^-1^. Cells were transferred to a microscope slide covered with 1.5% agarose and 1% glucose dissolved in 1×PBS. Cells were kept at 30°C and time-lapse movies were made at a frame rate of 10 min to follow the localization of MS2-GFP over time without the further presence of inducer. The disappearance of *bcaP_12bs_* mRNA was demonstrated by the loss of typical patterns of mRNA-bound MG4. Instead homogeneous distribution of MG4 in the cell was observed (**S1 Text - Fig 2J**). The homogenous distribution was either maintained or altered whenever *bcaP_12bs_* mRNA expression presumably became re-initiated due to induction by residual nisin present during the transfer from liquid medium to the agarose patch (data not shown).

1. **Plasmid and strain construction**

**Construction of *L. lactis* NZ9000 strains expressing MS2-GFP variants.** The gene encoding a superfolder GFP optimally performing in *L. lactis* [14] was amplified from pSEUDO::*Pusp45-sfgfp(Bs)* using primers pTE186 and pTE187, thereby extending the encoded protein at its N-terminal end with a flexible polylinker sequence (AGSGGEAEA). The amplified fragment and plasmid pNZ8048 were cleaved with *Pst*I and *Spe*I and ligated, yielding pLG-01. The *gfp* gene on pLG-01 was subjected to site-directed mutagenesis in order to obtain a monomeric version of GFP using primer set pLVG042A/pLVG043A. The amplified fragment was treated with USER^tm^ and used to transform competent cells of *L. lactis* NZ9000, yielding pLG-01m. Various MS2-GFP versions were constructed as follows: genes encoding full-length (MS2^wt^) and aggregation-deficient (MS2^ΔA^) MS2 variants were amplified from pZA25GFP [7], a kind gift of O. Amster-Choder, or pMS2-GFP [17], a kind gift from Robert Singer (Addgene plasmid #27121), respectively, using the primer set pMS2_FB_NcoI and pMS2_RB_PstI. A tandem dimer of full-length (MS2d^wt^) or aggregation deficient (MS2d^ΔA^) MS2 was obtained by amplification of their genes using primer sets MS2-FB-NcoI/MS2D-R-NheI or MS2D-F-NheI/MS2-RB-PstI, from pZA25GFP and pMS2-GFP, respectively, yielding MS2fl_up, MS2fl_down, MS2dA_up and MS2dA_down. These fragments were digested with *Nhe*I, after which MS2fl_up and MS2dA_up were ligated to MS2fl_down and MS2dA_down, respectively. The monomeric and dimeric *ms2* genes were subsequently cleaved with *Nco*I and *Pst*I and ligated into pLG_01m digested with the same enzymes, yielding the following plasmids: pLG-MG1, pLG-MG2, pLG-MG3, and pLG-MG4. See **S1 Text – Fig 2A** for an overview.

The P*_nisA_* promoter region followed by the gene of monomeric or dimeric aggregation-deficient MS2-GFP including two consecutive transcription terminators from pNZ8048, were concomitantly amplified from pLG-MG3 and pLG-MG4 using the primer pair pBvdZ001/pBvdZ002. The DNA fragments were cleaved with *Ava*I and *Bam*HI, ligated in properly digested pSEUDO10 (26) and introduced in *E. coli* DH5α, yielding pSEUDO10:*mg3* and pSEUDO10:*mg4*. Plasmid pSEUDO10:*mg3* or pSEUDO:*mg4* was introduced in the silent *pseudo10* locus of *L. lactis* NZ9000 by electrotransformation as previously described [16,18]. A second recombination step removed all plasmid sequences, leading to a clean, and single-copy knock-in of P*_nisA_-mg3* or P*_nisA_*-*mg4* in the chromosome *of L. lactis* NZ9000. The resulting strains, LG008 and LG010, produce, upon the addition of nisin A, an aggregation-deficient MS2 protein (MG3) or a dimerized and aggregation-deficient MS2 protein (MG4), each C-terminally fused to a monomeric superfolder GFP variant. The dimerized aggregation-deficient MS2-GFP fusion was selected for further use, and is referred to as MS2-GFP in the **main text**.

**Construction of *L. lactis* LG029 expressing DnaK-GFP.** A DNA fragment containing 1000 nts of the sequence upstream of the *dnaK* stop codon, and a second fragment covering 800 nts downstream of the *dnaK* stop codon were amplified from *L. lactis* MG1363 using primer sets pLVG163/pLVG164 and pLVG165/pLVG166, respectively. A DNA fragment encoding a flexible linker fused to monomeric superfolder GFP was amplified from pSEUDO10:*mg4* using primer pair pLVG060/pLVG61, while plasmid pCS1966, serving as the backbone vector, was amplified using primers pLVG072/pLVG073. All four fragments were purified, mixed, treated with the USER^tm^ enzyme mixture, and introduced in *E. coli* DH5α. The resulting plasmid, pCS1966-*dnaK-gfp,* was transferred to *L. lactis* NZ9000. A double cross-over recombination strategy based on 5-FOA counter-selection (19) yielded strain LG029, which contains a clean replacement of the original *dnaK* gene with *dnaK-gfp*.

**Construction of *L. lactis* LG045a expressing ParB-GFP.** The *parB* gene, originating from lactococcal plasmid pLP712 was amplified from pSEUDO39:*parB-mKate2* (J. Siebring, M. Kjos, J.W. Veening, personal communication) using primers pLVG117 and pLVG118. A 100-nts DNA region containing the constitutive promoter of *rnY*, P*_rnY_*, was amplified from the chromosome of *L. lactis* MG1363 using primer pair pLVG052b/pLVG119. A DNA fragment encoding a flexible linker fused to monomeric superfolder GFP was amplified from pSEUDO10:*mg4* with pLVG060 and pLVG061. The pSEUDO39 backbone was amplified from pSEUDO39:*mKate2* (J. Siebring, personal communication) with pLVG062 and pLVG063. Fragments were assembled via USER^tm^ treatment and introduced in *E. coli* DH5α cells, after which the correct plasmid, pSEUDO39:P*rnY-parB-gfp*, was obtained and inserted in the silent *pseudo39* locus of *L. lactis* NZ9000, yielding strain LG045a.

**Construction of *L. lactis* LG024a ectopically expressing RnY-GFP.** The *L. lactis* gene llmg_2156 (*rnY*) including 100 nts of its upstream region were amplified from *L. lactis* MG1363 chromosomal DNA using primer pair pLVG052 and pLVG053. For the construction of pSEUDO39:*rnY-gfp*, the pSEUDO39:*mKate2* plasmid was amplified with pLVG063 and pLVG062, while a DNA fragment encoding a flexible linker fused to monomeric superfolder GFP was amplified from pSEUDO10:*mg4* with pLVG060 and pLVG061. The three fragments were mixed, treated with the USER enzyme mixture, and used to transform *E. coli* DH5α cells. The obtained plasmid, pSEUDO39:*rnY-gfp*, was then introduced in the silent *pseudo39* locus of *L. lactis* NZ9000, resulting in strain LG024a.

**Nisin-inducible genes containing MS2 binding sites.** We used identical 5’ and 3’-sequences in all transcripts used in this study, which allowed specifically studying the role of the coding regions. The *bcaP* gene (*llmg_0118*; *ctrA*) was amplified from *L. lactis* MG1363 chromosomal DNA with primers pLVG017b (NcoI site overlapping the *bcaP* start codon) and pLVG018b (*Bam*HI site and a STREPII-tag coding sequence). The amplified fragment was restricted with *Nco*I and *Bam*HI and inserted immediately upstream of the 12 MS2 binding sites in pSL-MS2-12x, a gift from Robert Singer (Addgene plasmid #27119). The resulting vector was restricted with *Nco*I and *Spe*I, after which the DNA fragment containing the *bcaP* gene fused to the 12 MS2 binding sites was ligated into pNZ8048, resulting in pLG-BcaP. Plasmid pLG-BcaP was used to create pLG-PS1Δ9 and pLG-CodY as follows: A DNA fragment carrying *codY* (*llmg_0172*) was amplified from *L. lactis* MG1363 chromosomal DNA using primer pair pLVG028b/pLVG029b. The human *PS1Δ9* gene was amplified from pNZ-PS1Δ9 using primers pLVG019b and pLVG020b. Both fragment and pLG-BcaP were restricted with NcoI and BamHI. Ligation of each fragment seperately with digested pLG-BcaP and electrotransformation of *L. lactis* LG010 with the ligation mixtures yielded the plasmids pLG-PS1Δ9 and pLG-CodY. Plasmids pLG-SUT1 and pLG-GFP were constructed by uracil excision-based cloning. The backbone of pLG-BcaP, lacking the *bcaP* gene, was amplified using primers pLVG071 and pLVG106 for pLG-SUT1, and primers pLVG071 and pLVG121 for pLG-GFP. The genes *SUT1* or *gfp* were amplified from pNZ-StSUT1 or pSEUDO::*Pusp45-sfgfp(Bs),* using primer sets pLVG107/pLVG108 or pLVG120/pLVG122, respectively. The fragments were treated with USER^tm^ as described previously and introduced into electrocompetent *L. lactis* strain NZ9000 and/or LG010. pLG-BcaP-GFP and pLG-PS1Δ9-GFP were created as follows: A DNA fragment with a flexible linker followed by the gene for monomeric superfolder GFP was amplified from pSEUDO10:*mg4* using primer set pLVG060/pLVG121. Backbone DNA fragments were created using primer pair pLVG096/pLVG121 or pLVG145/pLVG121 with pLG-BcaP or pLG-PS1Δ9 as templates, respectively. Each amplified backbone fragment was mixed with the GFP gene-carrying DNA fragment, treated with USER^tm^ and introduced in *L. lactis* NZ9000. Plasmids pLG-2tmBcaP-PS1Δ9 and pLG-2tmPS1-BcaP were constructed by uracil excision-based cloning. The region in *bcaP* specifying the first two transmembrane domains of BcaP was amplified with primer pair pLVG143/pLVG144B from pLG-BcaP, while the gene fragment specifying PS1Δ9 lacking the first two transmembrane domains was obtained using primer pair pLVG139B/pLVG144B and pLG-PS1Δ9 as the template. The two DNA fragments were combined, treated with USER enzyme, and used to transform electrocompetent *L. lactis* NZ9000 cells, to yield pLG-2tmBcaP-PS1Δ9. To obtain pLG-2tmPS1-BcaP, the gene segment encoding the first two transmembrane domains of PS1Δ9 was amplified with primers pLVG140 and pLVG141 from pLG-PS1Δ9, while the backbone vector carrying the *bcaP* gene lacking the sequence for the first two transmembrane domains was amplified using primers pLVG139B and pLVG142B and the template plasmid pLG-BcaP. After ligation of both fragments, the correct plasmid was obtained in *L. lactis* NZ9000. pLG-BLS-PS1Δ9 as follows. The N-terminal domain of BcaP, including the first two transmembrane domains, was amplified with primers pLVG140 and pLVG146, while the pNZ9048 plasmid backbone containing the *PS1Δ9-12bs* gene was amplified using pLVG039b and pLVG149 from pLG-PS1Δ9. The fragments were annealed using USER cloning and introduced in *L. lactis* NZ9000 cells. The resulting plasmid contains a sequence encoding said N-terminal domain of BcaP, followed by a flexible linker followed by a TEV protease site (ENLYFQG) and PS1Δ9.

**Disruption of the ribosome binding sites on pLG-GFP, pLG-BcaP, and pLG-PS1Δ9.** The ribosome binding site (RBS; originating from the *nisA* gene) upstream of *gfp*_12bs_*, bcaP_12bs_* or *PS1Δ9_12bs_* on pLG-GFP, pLG-BcaP or pLG-PS1Δ9, respectively, was mutated from *aaggagg* into *aattcgg* by site-directed mutagenesis, using primer pair pLVG109/pLVG110, yielding pLG_ΔRBS-_GFP, pLG_ΔRBS_-BcaP, and pLG_ΔRBS_-PS1Δ9, respectively. The plasmids were picked up in *L. lactis* NZ9000.

**Introduction of the *parS* sequence on plasmids pNZ8048, pLG-BcaP and pLG-PS1Δ9.** A *parS* sequence (M. Kjos, J. Siebring, J. W. Veening, personal communication) was introduced in pNZ8048, pLG-BcaP, and pLG-PS1Δ9 by site-directed mutagenesis, using primers pJS89 and pJS90. Both primers contain a sequence extension that assembles into the functional *parS* sequence after annealing of the overhangs. The newly generated plasmids pNZ8048(*parS*), pLG(*parS*)-BcaP and pLG(*parS*)-PS1Δ9 were introduced in *L. lactis* LG045a.

1. **S1 Text - Figures**


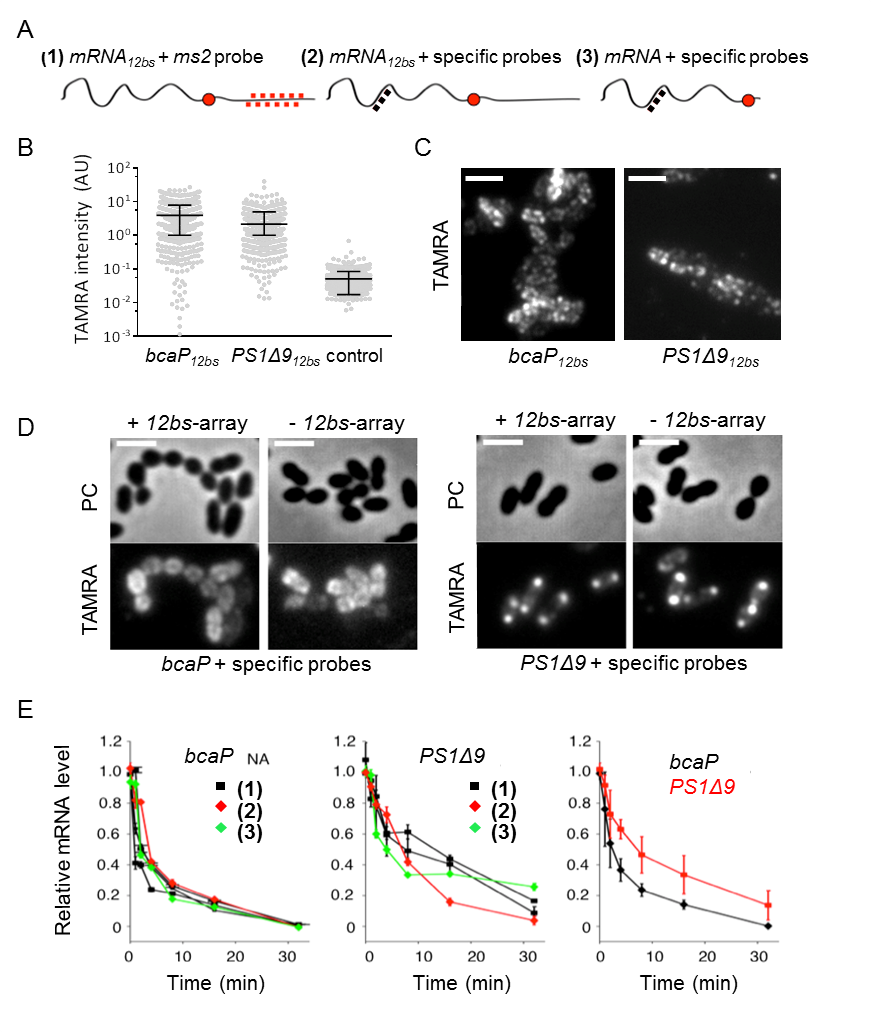


**S1 Text - Fig 1. Validation of FISH using *ms2* probes in combination with the *12bs-*RNA appendix to visualize overexpressed transcripts in *L. lactis.***

**(A)** Schematic representation of the combination of probes and annealing sites on the transcripts used to validate spFISH. Probes were designed to anneal to the *12bs*-array introduced at the 3’-end of the transcripts (1; red squares) or to gene-coding regions of the *bcaP* or *PS1Δ9 mRNAs* ((2) and (3); black squares). The red dot indicates the stop codon. All probes carried carboxytetramethylrhodamine (TAMRA)-labeled 5’ and 3’ ends. **(B)** Total TAMRA fluorescence levels in *bcaP* or *PS1Δ9*-expressing cells, as well as in control *L. lactis* NZ9000 cells. *bcaP* or *PS1Δ9* transcripts, each extended with the *12bs*-array, was overexpressed for 1 hr with 5 ng ml^-1^ nisin in cells that had reached the mid-exponential phase of growth. The induced cells, as well as uninduced control cells, were collected and mRNA levels inside individual cells were determined using spFISH in combination with fluorescence microscopy. **(C)** Exemplary fluorescence micrographs of *L. lactis* cells expressing either *bcaP_12bs_* (left panel) or *PS1Δ9_12bs_* (right panel) as visualized with spFISH. Exposure time: 1 sec. **(D)** Fluorescence micrographs of cells in which *bcaP* or *PS1Δ9*-specific probes were used to visualize overexpressed *bcaP* or *bcaP_12bs_*, or *PS1Δ9* or *PS1Δ9_12bs_*, respectively. Exposure time: 5 sec. PC = phase-contrast. White bar = 2 um. **(E)** Degradation rates of overexpressed *bcaP* or *PS1Δ9* after rifampicin addition, as monitored with FISH probes that target different sites of the transcripts (as depicted in **(A)**). The *ms2* probe targets the 3’-*12bs*-extension, while the specific probes are specific for *bcaP* or *PS1Δ9* mRNA. Gene-specific probes were used to follow the decay in time of *bcaP* mRNA (**(E)**; left panel) or *PS1Δ9* mRNA (**(E)**; center panel) with or without a 3’-*12bs*-extension (green and red diamonds, respectively): The degradation plots of the transcripts with or without the 3’-*12bs*-extension were similar for both pairs, indicating that the extension does not influence mRNA degradation. The use of the *ms2* probe for the quantification of mRNA degradation (black squares) yielded very comparable datasets. The degradation curves of *bcaP* mRNA (black squares) and *PS1Δ9* mRNA (red squares) from the experiments with different probes were combined and mean degradation with standard deviations were obtained and displayed in **(E)**, right panel.

**
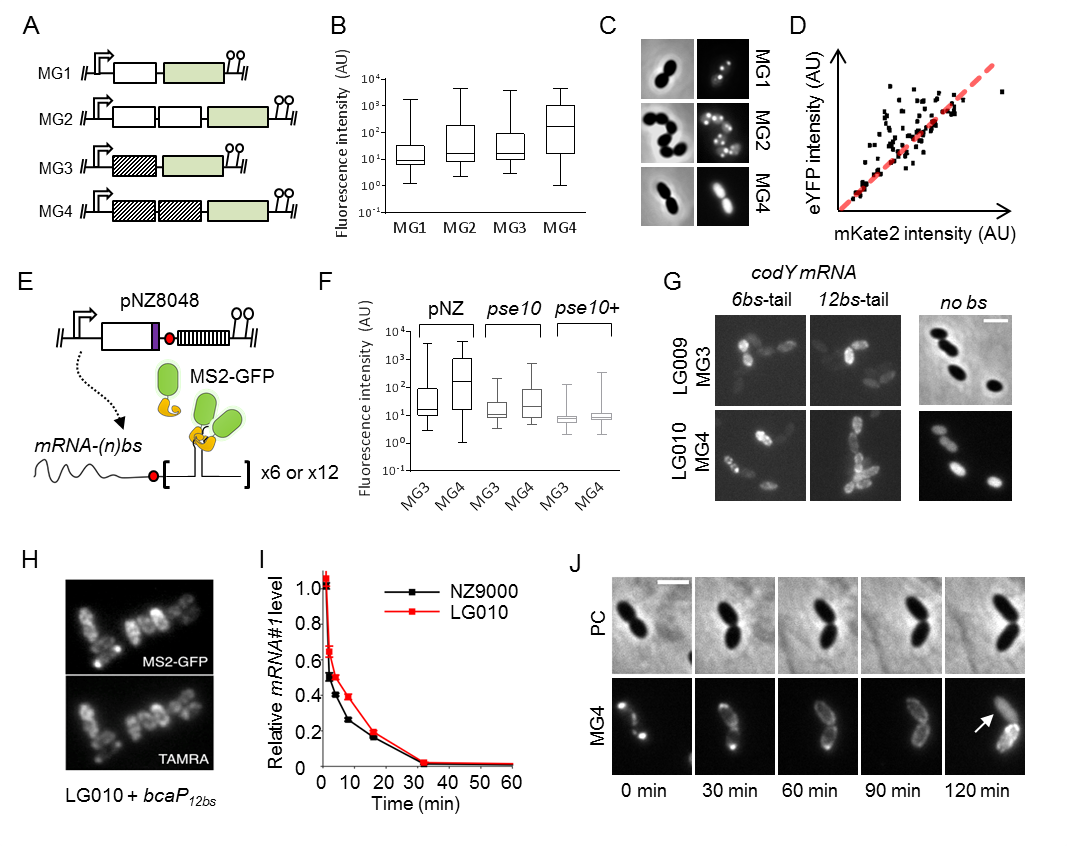
**

**S1 Text - Fig 2. Evaluation of the performance of overexpressed MS2-GFP variants in single cells of *L. lactis*.** **(A)** Schematic representation of the MS2-GFP variants used in this study. Besides the aggregation-prone full-length MS2 protein, MS2^wt^ (MG1), its dimerized version, MS2d^wt^ (MG2), and the assembly-deficient MS2 protein MS2^ΔA^ (MG3), a tandem dimer of the assembly-deficient MS2 variant was constructed, MS2d^ΔA^ (MG4). All of these MS2 proteins were fused, via a flexible linker, to a monomerized version of a superfolder GFP (sfGFP^m^; V206K substitution). The genes of each of these fusion proteins were placed under the nisin-controllable *L. lactis* promoter (P*_nisA_*) (right-bent arrow) and were followed by two consecutive transcriptional terminators (lollipops). Green rectangle, *gfp* gene; White rectangle, full-length *ms2* gene; Hatched rectangle, aggregation-deficient *ms2* gene. **(B)** Single-cell distributions of GFP fluorescence after 1hr overexpression of the four MG variants using fluorescence microscopy. **(C)** Fluorescence micrographs of cells expressing aggregation-prone MG1 or MG2, and aggregation-resistant MG4. Fluorescence microscopy revealed that extended (>1hr) and high (>5 ng ml^-1^ nisin) expression of MS2^wt^ proteins in *L. lactis* NZ9000 resulted in MS2-GFP aggregation. **(D)** The level of intracellular mKATE2 fluorescence as a function of the intracellular eYFP fluorescence in *L. lactis* NZ9000. Both fluorescent proteins were placed under the control of the P*_nisA_*, but mKATE2 was expressed from a pIL252 vector, while eYFP was expressed from pNZ8048. As these plasmids are compatible, they can both be maintained in the same *L. lactis* cell. The fluorescence intensities of mKATE2 and of eYFP per cell show a high correlation, indicating that the transcription ratio between the two expression modules is constant in each cell. **(E)** Representation of the experimental set-up of MS2-based mRNA-tagging in *L. lactis.* An array of 12 MS2 binding sequences (striped box, abbreviated to _12bs_) was introduced behind the stop codon (red dot) at the 3’ UTR of the studied transcripts (white box) that were extended with a 24-nt sequence coding for StrepII-tag (AWSHPQFEK; purple box) to solely visualize fully transcribed mRNA molecules. To account for expression heterogeneity within the population, both an MS2-GFP variant and target RNA were placed under control of P*_nisA_* (right turn arrow). In this way, the single-cell expression ratio of MS2-GFP protein to target mRNA was kept similar (data not shown). **(F)** Fluorescence intensities of *L. lactis* NZ9000 cells expression MG3 or MG4 from pNZ8048 (pNZ), *L. lactis* LG009 or LG010 cells expressing MG3 or MG4 from a chromosomal locus (*pse10*), respectively, or *L. lactis* LG009 or LG010 cells expressing MG3 or MG4 and at the same time overexpressing target mRNA from pNZ8048 (*pse10+*). In all cases, transcription was induced with 5 ng ml^-1^ nisin for 1 hr, after which single-cell fluorescence (given as arbitrary units (AU) on the Y-axis) of cells in the population was measured using fluorescence microscopy. **(G)** In *L. lactis* strains LG009 and LG010, co-expression of MG3 or MG4, respectively, with *codY* mRNA extended with either 6 (*6bs*) or 12 (*12bs*) MS2 binding sites from the multi-copy pNZ8048 plasmid yielded typical fluorescence localization patterns, a representative example of which is given in these fluorescence micrographs. In the absence of transcripts with MS2 binding sites, MG4 is homogeneously distributed throughout the cell (lower right-most panel). **(H)** Fluorescence images depicting the localization patterns in *L. lactis* LG010 of *bcaP_12bs_* captured simultaneously with the MS2 system and with spFISH (TAMRA). **(I)** Degradation curves of *bcaP_12bs_* mRNA in the presence (strain LG010) or absence (strain NZ9000) of MG4 protein. Mid-exponential-phase NZ9000 or LG010 cultures were induced with 5 ng ml^-1^ nisin to express *bcaP_12bs_* mRNA for 1 hr, after which rifampicin was added to the cultures. Time-point samples were drawn from the cultures, and the average mRNA levels of the samples were determined using spFISH, fluorescence microscopy, and normalized to the average mRNA level at the first sample (t =0). **(J)** Snap-shots from fluorescence microscopy observations of the spatiotemporal behavior of *bcaP_12bs_* mRNA-bound by MG4 in *L. lactis* LG010*.* Expression of *bcaP_12bs_* mRNA and MG4 was induced for 1 hr by the addition of nisin to a final concentration of 5 ng ml^-1^. Subsequently, cells were transferred to a microscope slide covered with 1.5% agarose and 1% glucose dissolved in 1×PBS. Cells were kept at 30°C and time-lapse movies were taken (frame rate: 10 min) to follow the localization of MG4 over time in the absence of inducer. All scale bars in microscopy images represent 2 µm.

1. **S1 Text - References**

1. Raj A, van Oudenaarden A. Single-molecule approaches to stochastic gene expression. Annu Rev Biophys. 2009;38: 255–70. doi:10.1146/annurev.biophys.37.032807.125928

2. Stoecker K, Dorninger C, Daims H, Wagner M. Double Labeling of Oligonucleotide Probes for Fluorescence In Situ Hybridization (DOPE-FISH) Improves Signal Intensity and Increases rRNA Accessibility. Appl Environ Microbiol. American Society for Microbiology; 2010;76: 922–926. doi:10.1128/AEM.02456-09

3. de Ruyter PG, Kuipers OP, de Vos WM. Controlled gene expression systems for Lactococcus lactis with the food-grade inducer nisin. Appl Environ Microbiol. 1996;62: 3662–7.

4. Skinner SO, Sepúlveda LA, Xu H, Golding I. Measuring mRNA copy number in individual Escherichia coli cells using single-molecule fluorescent in situ hybridization. Nat Protoc. 2013;8: 1100–13. doi:10.1038/nprot.2013.066

5. Montero Llopis P, Jackson AF, Sliusarenko O, Surovtsev I, Heinritz J, Emonet T, et al. Spatial organization of the flow of genetic information in bacteria. Nature. 2010;466: 77–81. doi:10.1038/nature09152

6. Golding I, Cox EC. RNA dynamics in live Escherichia coli cells. Proc Natl Acad Sci U S A. 2004;101: 11310–5. doi:10.1073/pnas.0404443101

7. Nevo-Dinur K, Nussbaum-Shochat A, Ben-Yehuda S, Amster-Choder O. Translation-independent localization of mRNA in E. coli. Science. 2011;331: 1081–4. doi:10.1126/science.1195691

8. dos Santos VT, Bisson-Filho AW, Gueiros-Filho FJ. DivIVA-mediated polar localization of ComN, a posttranscriptional regulator of Bacillus subtilis. J Bacteriol. 2012;194: 3661–9. doi:10.1128/JB.05879-11

9. Golding I, Paulsson J, Zawilski SM, Cox EC. Real-time kinetics of gene activity in individual bacteria. Cell. 2005;123: 1025–36. doi:10.1016/j.cell.2005.09.031

10. Valegård K, Murray JB, Stockley PG, Stonehouse NJ, Liljas L. Crystal structure of an RNA bacteriophage coat protein�operator complex. Nature. Nature Publishing Group; 1994;371: 623–626. doi:10.1038/371623a0

11. Ni C-Z, Syed R, Kodandapani R, Wickersham J, Peabody DS, Ely KR, et al. Crystal structure of the MS2 coat protein dimer: implications for RNA binding and virus assembly. Structure. Elsevier; 1995;3: 255–263. doi:10.1016/S0969-2126(01)00156-3

12. Wu B, Chao J a, Singer RH. Fluorescence fluctuation spectroscopy enables quantitative imaging of single mRNAs in living cells. Biophys J. Biophysical Society; 2012;102: 2936–44. doi:10.1016/j.bpj.2012.05.017

13. Peabody DS, Ely KR. Control of translational repression by protein-protein interactions. Nucleic Acids Res. Oxford University Press; 1992;20: 1649–55.

14. Overkamp W, Beilharz K, Detert Oude Weme R, Solopova A, Karsens H, Kovacs AT, et al. Benchmarking Various Green Fluorescent Protein Variants in Bacillus subtilis, Streptococcus pneumoniae, and Lactococcus lactis for Live Cell Imaging. Appl Environ Microbiol. 2013;79: 6481–6490. doi:10.1128/AEM.02033-13

15. King MS, Boes C, Kunji ERS. Membrane protein expression in Lactococcus lactis. Methods in enzymology. 2015. pp. 77–97. doi:10.1016/bs.mie.2014.12.009

16. Pinto JPC, Zeyniyev A, Karsens H, Trip H, Lolkema JS, Kuipers OP, et al. pSEUDO, a genetic integration standard for Lactococcus lactis. Appl Environ Microbiol. 2011;77: 6687–90. doi:10.1128/AEM.05196-11

17. Fusco D, Accornero N, Lavoie B, Shenoy SM, Blanchard JM, Singer RH, et al. Single mRNA molecules demonstrate probabilistic movement in living mammalian cells. 2003;13: 161–167. doi:10.1016/S0960-9822(02)01436-7

18. Solem C, Defoor E, Jensen PR, Martinussen J. Plasmid pCS1966, a new selection/counterselection tool for lactic acid bacterium strain construction based on the oroP gene, encoding an orotate transporter from Lactococcus lactis. Appl Environ Microbiol. 2008;74: 4772–5. doi:10.1128/AEM.00134-08
